# Network evaluation of an innovation platform in continuous quality improvement in Australian Indigenous primary healthcare

**DOI:** 10.1186/s12961-022-00909-z

**Published:** 2022-10-31

**Authors:** Frances Clare Cunningham, Boyd Alexander Potts, Shanthi Ann Ramanathan, Jodie Bailie, Roxanne Gwendalyn Bainbridge, Andrew Searles, Alison Frances Laycock, Ross Stewart Bailie

**Affiliations:** 1 Wellbeing and Preventable Chronic Diseases Division, Menzies School of Health Research, Charles Darwin University, Brisbane, QLD Australia; 2 Health Research Economics, Hunter Medical Research Institute, Newcastle, NSW Australia; 3 College of Health, Medicine and Wellbeing, The University of Newcastle, Newcastle, NSW Australia; 4 University Centre for Rural Health, The University of Sydney, Lismore, NSW Australia; 5 School of Public Health, The University of Sydney, Sydney, NSW Australia; 6 Poche Centre for Indigenous Health, The University of Queensland, Toowong, QLD Australia; 7 Faculty of Medicine and Health, The University of Sydney, Sydney, NSW Australia

**Keywords:** Coalition, Collaborative, Evaluation, Indigenous health, Innovation platform, Primary healthcare, Quality improvement, Social network analysis

## Abstract

**Background:**

From 2014 to 2019, the Centre for Research Excellence in Integrated Quality Improvement (CRE-IQI) was evaluated as an innovation platform focusing on continuous quality improvement in Indigenous Australian primary healthcare. Although social network analysis (SNA) is a recognized method for evaluating the functioning, collaboration and effectiveness of innovation platforms, applied research is limited. This study applies SNA to evaluate the CRE-IQI’s functioning as an innovation platform.

**Methods:**

Two surveys (2017, 2019) were conducted using social survey and network methods. Survey items covered respondent characteristics, their perceptions of the CRE-IQI’s performance, and its impact and sociometric relationships. Members’ relationship information was captured for the CRE-IQI at three time points, namely start (retrospectively), midpoint and final year, on three network types (knew, shared information, collaborated). SNA software was used to compute standard network metrics including diameter, density and centrality, and to develop visualizations. Survey and network results were addressed in a workshop held by members to develop improvement strategies.

**Results:**

The response rate was 80% in 2017 and 65% in 2019 (*n* = 49 and 47, respectively). Between 2017 and 2019, respondents’ mean ratings of the CRE-IQI’s functioning and achievements in meeting its goals were sustained. They perceived the CRE-IQI as multidisciplinary, having effective management and governance, and incorporating Indigenous research leadership, representation and ways of working. Respondents recognized high levels of trust amongst members, rated “good communication and coordination with participants” highly, and “facilitating collaboration” as the CRE’s most strongly recognized achievement. In collaboration and information-sharing networks, average path length remained low in 2017 and 2019, indicating good small-world network properties for relaying information. On average, respondents shared information and collaborated with more CRE members in 2017 than 2019. However, in both 2017 and 2019 there were new collaborations and information-sharing outside of direct collaborations. CRE-IQI outcomes included: evidence generation; knowledge transfer and skills development in quality improvement; research capacity-building, career development; mentoring; grant support; development of new projects; health service support; and policy impact.

**Conclusions:**

This study shows the utility of network analysis in evaluating the functioning, and collaboration, at the individual, organizational and health system levels, of an innovation platform, and adds to our understanding of factors enabling successful innovation platforms.

**Supplementary Information:**

The online version contains supplementary material available at 10.1186/s12961-022-00909-z.

## Background

Strengthening primary healthcare (PHC) systems is vital to improving health outcomes and reducing inequity [1–3]. The disparities in health status and inequitable access to healthcare for Aboriginal and Torres Strait Islander Australians (hereafter, respectfully referred to collectively as Indigenous Australians) compared with the rest of the Australian population are widely recognized [4]. The Centre for Research Excellence in Integrated Quality Improvement (CRE-IQI) was funded by the Australian National Health and Medical Research Council to operate from November 2014 to November 2019 as an innovation platform for systems-wide improvement in Indigenous PHC. It aimed to foster collaborations between researchers, service providers and policy-makers for priority-driven research and implementation, thereby strengthening state-of-the-art quality improvement systems in Indigenous PHC across Australia. The CRE-IQI drew on international experience of innovation platforms to expand the partnership learning model established through the predecessor research entity, the ABCD National Research Partnership, and other work associated with the CRE-IQI [5–8].

Innovation platforms are becoming internationally recognized as a collaborative mechanism for bringing together stakeholders to identify solutions to common problems or achieve common goals [9, 10]. Purposefully open to the entry of new members bringing new competencies, they seek to maximize contributions from a varied knowledge base while achieving coherence by having minimal hierarchy [11]. Internationally, innovation platforms have been used to organize and coordinate distributed innovation processes with high degrees of complexity [11]. They emerged as a departure from the historical linear approach to agricultural extension programs [12], influenced through the application of innovation systems ideas [13] to the agricultural research arena [14], and providing evidence of positive impact [15, 16]. Innovation platforms have been applied in different fields, including health, to a limited extent [17–21]. For example, McHugh et al. found that multi-stakeholder alliances, a form of innovation platform, may encourage uptake of information technology in medical practices to improve quality [21].

Innovation platforms influence innovation through multiple levels and pathways. This has implications for how they need to be organized internally to coordinate multiple actors and changes simultaneously. Key activities that are critical to achieving impact include facilitating and establishing communication practices among stakeholders; aligning with government policies; capacity-strengthening of stakeholders; building common ground and networks among stakeholders; and planning formal structural activities to deliver impact [22].

Innovation platforms are a type of network. They are designed to ensure inclusivity of various groups to promote interactions (represented by the edges in the network) among actors (the nodes in the network) by building the network to promote knowledge-sharing and collaboration, and to facilitate diffusion of innovation [23]. Social network analysis (SNA) can be used to examine structural relationships, influence and information flows in networks, and network sustainability [24]. There is evidence across sectors, including healthcare [25], that effective networks employ natural structural features (e.g. bridges, brokers, density, centrality, degrees of separation, social capital, trust) to produce collaborative work [26]. Such collaboration requires efficient sharing of information, and social and professional interaction within and across networks. Various authors have drawn attention to the potential of such networks in securing desirable outcomes in healthcare and elsewhere [27–29].

### Conceptual framework for evaluation

The conceptual framework for this evaluation includes application of the life cycle approach, the assessment of network governance and management, and the application of network metrics to examine communication, information-sharing and collaboration in the innovation platform. The temporal aspect of network development is reflected in three main life cycle stages: the initial stage of development, the developed network and the mature network [30]. Provan and Kenis explored the impact of governance and the role of management on network effectiveness [31, 32]. The latter is associated with the importance of achieving network goals [33] and the capacity for innovation and change [33]. Various authors informed the application of network metrics to examine network topology, connectivity and diffusion of innovations [30, 34–37].

The literature provided guidance on the central aspects of innovation platforms that should be captured in an evaluation. Kilelu et al. identified the need for more research on the governance mechanisms of innovation platforms and on monitoring systems that could help platform members and facilitators adjust to changing needs [38]. Cadilhon proposed an evaluation framework that examines the structure, conduct and performance of the innovation platform, advocating that the framework be used at various stages in the life of an innovation platform to measure improvement in the collaborative conduct of stakeholders and progress towards achieving the objectives of the platform [39, 40]. Schut et al. showed the utility of SNA in measuring the performance of innovation platforms in agricultural development research, and also supported its use in ongoing mapping of the evolution of such networks over time [41]. In addition to these network aspects, researchers have identified that building stakeholder capability is important for sustaining innovation platforms and for equipping stakeholders to take on future challenges [11, 42].

### Aim of study

This study aimed to use survey and SNA methods to monitor and evaluate how well the CRE-IQI worked as an innovation platform over its life span and to capture changes in the CRE-IQI network. To our knowledge, innovation platforms have not previously been used in Australian health research, healthcare settings or Indigenous PHC. The CRE-IQI provided the opportunity to evaluate the utility of applying an innovation platform in this context in Australia. Although SNA is recommended as a key evaluation method for studying the functioning and impacts of innovation platforms [43], there is a paucity of published research on such applications [39]. This study addresses this gap. It contributes to a wider evaluation of the CRE-IQI which included developmental evaluation and impact evaluation [5, 44–48], and to literature on innovation platforms [22] and applied research partnerships and collaborations [6].

## Methods

### Study setting

**Fig. 1:**
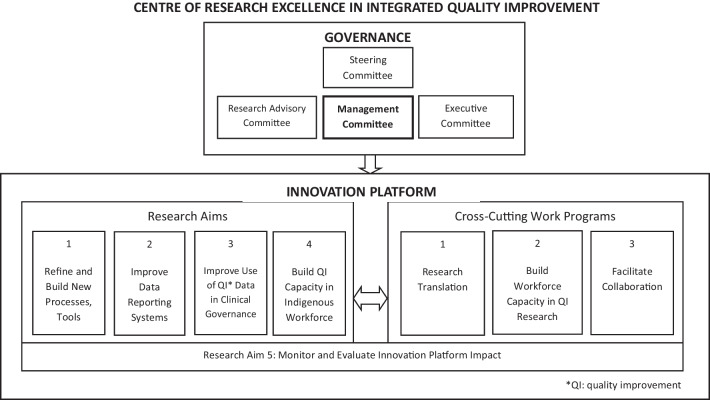
Governance and management of the CRE in IQI

The CRE-IQI’s organizational structure is shown in Fig. 1. Governance was carried out by a management committee representing key stakeholders (primarily CRE-IQI members with diverse expertise). They included Indigenous and non-Indigenous members in New South Wales, Queensland and the Northern Territory who had roles in universities, peak Indigenous regional bodies and government. An executive committee, supported by the coordinating centre, managed day-to-day operations and reported to the management committee. The coordinating centre provided support for all CRE-IQI work programs.

The goals of the CRE-IQI were to (1) refine and build new processes and tools; (2) improve data reporting systems; (3) improve the use of quality improvement data in clinical governance, management and practice; (4) build quality improvement capacity in the Indigenous workforce and (5) monitor and evaluate the impact of the CRE-IQI. There were three cross-cutting work programs to (1) promote the transfer of research outcomes into health policy and practice, (2) develop the capacity of the health and medical research workforce and (3) facilitate collaboration. The CRE-IQI built on the membership of the predecessor research entity, the ABCD National Research Partnership, and was open to new membership from the wider networks of current members and their organizations [7]. The CRE-IQI actively invited new membership on a nationwide basis from organizations and agencies involved in quality improvement research in Indigenous PHC. The CRE-IQI held biannual face-to-face meetings, with research masterclasses conducted in association with the meetings. Online monthly research capacity-building seminars were also held. Seed funding and other support was available for emerging research projects, and scholarship support for postgraduate students. Further details about how the CRE-IQI operated as an innovation platform are published elsewhere [5, 45, 48, 49].

### Study approach

An evaluation group guiding the overall evaluation of the CRE-IQI provided direction on the study design and methods, which were then presented and discussed with members at successive biannual face-to-face meetings of the CRE-IQI. Feedback and advice were incorporated into the study design and methods. A desktop review of existing documentation, materials and records of the CRE-IQI was undertaken to gain an understanding of its formation, governance, management and operation.

The first cross-sectional online survey (Survey 1) of members, conducted from December 2017 to January 2018, captured data for two points in time—retrospectively for the commencement of the CRE-IQI and at midpoint. Survey 2 was conducted from May to July 2019 in the final year of the CRE-IQI. Current literature informed the development of survey items [30, 32–37]. The surveys captured member demographic information, members’ perceptions on the functioning of the CRE-IQI from a network governance and management perspective, and information on social network relationships. For the network relationship data, respondents were asked to identify from the list of CRE-IQI members those (1) whom they knew prior to its establishment, (2) to whom they provided information or advice relating to the CRE and its work, (3) from whom they received information or advice relating to the CRE and its work, and (4) with whom they collaborated on CRE-related research or a CRE-related project. Hence, the CRE-IQI network structure could be examined at its start-up, at its midpoint, and in the final year. Copies of both surveys are included as Additional file 1 and Additional file 2.

### Participants and recruitment

The study used the form of SNA termed a “whole-network” approach [50] to examine the relationships or ties between members involved in the CRE-IQI. For each survey, a “bounded list” of current members (i.e. setting the boundary around the set of actors to be included [51]) was provided by the CRE-IQI coordination centre from which the survey sample was drawn. Criteria for the inclusion of “active members” in the sample were developed with the evaluation group. Inclusion criteria required active participation in the CRE-IQI in the previous 12 months and included chief and associate investigators, management committee, steering committee and research advisory committee members, current employees of the CRE-IQI, those receiving research funding from the CRE-IQI or involved in key project groups, co-authors on CRE-IQI peer-reviewed publications and attendees at CRE-IQI meetings. As strategies were introduced to encourage wider engagement following feedback from Survey 1, an additional “snowball” step was used in Survey 2 to ensure that the whole reach of the network could be captured. Recipients were asked to nominate additional participants of the CRE-IQI with whom they had worked on CRE-IQI-related initiatives during the previous 12 months, who were not listed in the survey sample. The survey was then sent to these additional people, who were also asked to nominate others involved in the work. Participants nominated through the snowball process responded to a shortened version of the survey, comprising only the demographic and SNA items.

### Data collection and analysis

The online surveys were administered on two platforms, SurveyMonkey (Survey 1) and Qualtrics (Survey 2). An email invitation and a link to the survey were sent to 61 individuals for Survey 1 and 72 for Survey 2 (54 on the initial list; 18 from the snowball step). Email reminders were sent at 2-week intervals until the closing of the surveys, with telephone follow-up as required.

The four-item Likert scale perception data were assessed through self-ratings provided by survey respondents. For the demographic and non-network data, descriptive analysis was applied. Responses from each open-ended question were reviewed by FC and BP. For the open-ended question about the “most significant change” that the CRE-IQI had made for respondents, response categories reflecting the key themes were identified by FC and BP from the open-ended responses. All responses were entered into an Excel spreadsheet and allocated to a matching category, and each category was ranked from highest to lowest (1 to 4) based on response frequency. Network data were analysed using SNA methods [52] to assess the innovation platform from a network perspective. A summary of network terms and definitions is provided as Additional file 3: Table S1.

Data were manipulated (i.e. data were cleaned and survey output data were reshaped into node and edge table format to allow for network analysis) and analysed using the Python 3.7.4 programming language [53] and in the IBM SPSS Statistics for Windows, version 25.0 software. Two software packages were used for the network analysis and visualization, the Python package within NetworkX, and open-source Gephi software [54, 55]. Network analysis used NetworkX, which has a wide range of available algorithms for analysis. Being code-based (Python), it provided superior flexibility and efficiency in interrogating graph data in the exploratory phase of analysis compared with the point-and-click interface of the Gephi software. However, Gephi’s graph visualization features provide a larger number of graph layout algorithms and greater interactivity for achieving the best aesthetic properties for presentation. Gephi was used to develop network metrics, such as diameter, density and centrality, and to map the networks visually. The Gephi SNA tools provided graphic visualization of the structure of the network, including the position of members and their connections.

For the network analysis, it was possible to deduce edge data from existing respondent data for nonrespondents. For example, if a respondent reported knowing or collaborating with a nonrespondent, then that edge was also associated with the nonrespondent. For the sharing network, if a respondent reported receiving information from, or providing information to, a nonrespondent, then the nonrespondent was assumed to have provided or received that information, respectively, and the edge was allocated. This was computationally achieved by transposing the relevant vector from the adjacency matrices. Where the presence of an edge between two actors was not mutually confirmed, a positive bias was applied to include the edge. In Survey 2 it was not possible to deduce edge data for the snowball nonrespondents.

For the analysis of the network relationship data, each respondent was associated with a data vector in which a value of 1 represented the existence of a relationship (edge) between other network actors, and zero otherwise. Taken together, these vectors comprise the adjacency matrix: for example, for *g* actors, this will be a *g* × *g* array of edge information for network analysis. Adjacency matrices were compiled for the three networks—prior knowledge, information-sharing and collaboration.

In network analyses, degree distributions (the distribution of connections per node in the network) can be appraised for their shape and whether measures of central tendency (median, mean) can suitably represent the connectivity of the network, or whether they follow an exponential or power-law distribution. Power-law distributions are referred to as “scale-free” since values are distributed over a wide range, and measures such as the mean cannot be interpreted as the typical or expected value. In such distributions, network connectivity is driven predominantly by a few key nodes that have a high degree. Degree distributions were examined for each network.

Degree centralization was examined for each network. Centralization is a measure of the connectedness around the most central node (defined in this study by the highest node degree). It is denominated by the theoretical maximum generated by a star graph (i.e. a single central node to which each of the others is exclusively connected) of the same number of nodes. Values of 1 represent maximum centralization. Community detection was computed and analysed for statistical associations with organization type and primary work role. Effect sizes were examined using Cramér’s V.

Edge information (i.e. number of connections between actors/nodes) for both surveys was analysed using three contingency tables covering pairwise combinations of prior knowledge (of other members), information-sharing and collaboration. Statistical significance was tested using chi-square tests. Where sharing of information or collaboration occurred between two people who previously knew each other, this was coded as “exploit shares” or “exploit collaborations”, having capitalized on existing relationships. Conversely, sharing of information or collaboration between people who did not know each other prior to the CRE-IQI was coded as “new shares” or “new collaborations” and was considered an indication of success in the innovation platform. Collaborations that involved the direct sharing of information were coded as “strong collaborations” and those that did not involve direct sharing of information were coded as “weak”. Sharing of information outside of direct collaboration was coded as “network support” and was also considered an indication of success in the innovation platform.

### Review and strategy development

An interactive workshop was facilitated at the biannual face-to-face meeting of the CRE-IQI in May 2018 to encourage discussion of the findings from Survey 1. Participants were asked to consider insights and strategies to sustain CRE-IQI strengths identified in the report, and to address any areas that might need improvement. Recommendations were forwarded to the CRE-IQI management committee to address implementation. At the CRE-IQI biannual meeting in October 2019, findings from the two surveys were presented for discussion, feedback and reflection with members.

The authors of this paper were all involved as members of the CRE-IQI and completed surveys, and three of the authors (FC, JB, RB) were involved in management and coordination of the CRE-IQI. While the insights of these key CRE-IQI members contributed to the research design, understanding of the data and interpretation of study results, the use of objective network metrics helped mitigate the risk of potential study bias.

## Results

### Respondents’ characteristics and CRE-IQI involvement

There were 49 respondents to Survey 1 (61 invitations, 80.3% response rate), with 48 responding to the network items, and edge data deduced for an additional 13 nonrespondents. There were 47 respondents (40 original respondents and 7 through the snowballing process) to Survey 2 (72 invitations, 65% response rate). Edge data were deduced for an additional 18 nonrespondents. Response rates differed on some reported items.

As shown in Table 1, for both surveys, the largest proportion of respondents worked in a university or research organization, had a researcher role, were female and were over 40 years of age. There was an increase in the number of Indigenous respondents in Survey 2 (from 5 to 7). In both surveys, nearly half of the respondents reported previous involvement with the ABCD National Research Partnership, and more than half had been in their present work position for more than 2 years. Reflecting the CRE-IQI life cycle, in 2017, 53% had more than 2 years of involvement, while in 2019 that proportion increased by 11 percentage points to 64%.

**Table 1:** Member characteristics, 2017 and 2019

Respondent characteristics	2017		2019	
	Freq	%	Freq	%
*Workplace*	*n = 61*^*a*^	*100%*	*n = 47*	*100%*
University or research institute	41	67.2	32	68.1
Indigenous community-controlled health service	5	8.2	4	8.5
Indigenous community-controlled sector support organization (peak body)	7	11.5	4	8.5
Government-operated health service	4	6.6	2	4.3
Government health department	2	3.3	1	2.1
Other governmental organization	1	1.6	0	0.0
Other nongovernmental organization	1	1.6	0	–
Not available	0	0.0	4	8.5
*Gender*	*n = 61*^*a*^	*100%*	*n = 47*	*100%*
Female	49	80.3	33	70.2
Male	12	19.7	12	25.5
Not available	0	0.0	2	4.3
*Age*	*n = 61*^*a*^	*100%*	*n = 47*	*100%*
25–39 years	9	14.7	9	19.1
40+ years	40	65.6	36	76.6
Not available	12	19.7	2	4.3
*Primary work position*	*n = 61*^*a*^	*100%*	*n = 47*	*100%*
Researcher	36	59.0	28	59.6
Manager or administrator	8	13.1	2	4.3
Quality improvement facilitator	7	11.5	3	6.4
Medical practitioner/health practitioner	3	4.9	5	10.6
Student (PhD, Master’s, postgraduate)	3	4.9	3	6.4
Knowledge translation/evaluation	2	3.3	0	0.0
Indigenous health worker/practitioner	1	1.6	0	0.0
Policy or planning officer	1	1.6	1	2.1
Other staff	0	0.0	3	6.4
Not available	0	0.0	2	4.3
*Length of time in present position*	*n = 49*	*100%*	*n = 47*	*100%*
< 6 months	1	2.0	3	6.4
6 months–2 years	17	34.7	9	19.1
2–5 years	14	28.6	12	25.5
> 5 years	17	34.7	21	44.7
Not available	0	0.0	2	4.3
*Indigenous status*	*n = 49*	*100%*	*n = 47*	*100%*
Aboriginal	5	10.2	6	12.8
Torres Strait Islander	0	0.0	1	2.1
Aboriginal and Torres Strait Islander	0	0.0	0	0.0
Non-Indigenous	44	89.8	38	80.9
Not available	0	0.0	2	4.3
*Involved with ABCD*	*n = 49*	*100%*	*n = 47*	*100%*
Yes	21	42.9	20	42.6
No	28	57.1	18	38.3
Don’t know	0	0.0	1	2.1
Not available	0	0.0	8	17.0
*Length of involvement with CRE-IQI*	*n = 49*	*100%*	*n = 47*	*100%*
< 6 months	0	0.0	2	4.3
6 months–1 year	6	12.2	4	8.5
1–2 years	17	34.7	3	6.4
> 2 years	26	53.1	30	63.8
Not available	0	0.0	8	17.0

^a^Where *n*=61 (2017) characteristics of nonrespondents were inferred through publicly available information. Final project reporting deadlines did not permit such additional data retrieval in 2019

Based on 2019 data, 40% of respondents (*n* = 45) were involved in the CRE-IQI because of their previous involvement with the ABCD program [8]. More than half of the respondents (53%; *n* = 43) cited the ability to participate in the CRE-IQI’s professional and collaborative network as the main motivation for their participation. For example, one respondent remarked:

*I really value the national network of people from a wide range of disciplines who are all interested in building the knowledge base and practical application of quality improvement in Aboriginal PHC. It is inclusive and people bring knowledge and expertise from many different perspectives*.

Almost all respondents (Survey 1: 92% (*n* = 48); Survey 2: 97% (*n* = 39)) reported that their involvement in the CRE-IQI had assisted them in their research or in their health service. Respondents to an open-ended question identified the “most significant change” [56] that the CRE-IQI had made at each of four levels—for themselves, for their team/work group, for their Indigenous PHC service and for the wider system level (Additional file 3: Table S2). In both years, at the first three levels, increased networking/collaboration was most frequently identified, followed by knowledge transfer and skills in continuous quality improvement (CQI)/capacity-building. In 2017, at the wider system level, research translation and increased networking/collaboration were frequently identified, while in 2019, knowledge transfer and skills in CQI/capacity-building was reported most frequently, followed by policy impact, and then providing research evidence on CQI.

### Perceptions of CRE-IQI functioning

Additional file 3: Table S3 shows the respondents’ ratings of the CRE-IQI’s achievement in meeting its goals and functioning. In all areas of functioning and achievement, the mean ratings were sustained from 2017 to 2019, indicating that the functioning and achievements of the CRE-IQI were sustained over its life cycle (not tested for statistical significance). For both surveys, respondents rated the CRE-IQI most highly for its achievement in “facilitating collaboration”, followed by “monitoring and evaluating the impact of the CRE-IQI”, “improving use of quality improvement data in clinical governance, management and practice”, and “developing the capacity of the health and medical research workforce”. For CRE-IQI functioning, having “clear leadership of the CRE-IQI” was rated highest and usefulness of CRE-IQI materials (publications and reports) was rated lowest in both years.

### Network structure and features of innovation platform

Table 2 provides a summary of network values and other key metrics for both surveys.

**Table 2:** Summary of network metrics

Measure	Network type					
	Knew		Shared		Collaborated	
	2017	2019	2017	2019	2017	2019
Directed network	Undirected	Undirected	Directed	Directed	Undirected	Undirected
Nodes	61	65	61	65	61	65
Edges	513	509	1241	1194	461	447
Average degree	16.82 (median = 14)	15.66 (median = 14)	20.34 (median = 16)	18.37 (median = 14)	15.12 (median = 11)	13.75 (median = 9)
Network diameter	3	5	2	4	2	4
Density	0.28	0.25	0.34	0.29	0.25	0.22
Connected components	1	1	1	2	1	3
Average clustering	0.67	0.63	0.72	0.7	0.74	0.66
Average path	1.75	1.94	1.66	1.76	1.75	1.88
Reciprocity (sharing)	*NA*	*NA*	0.84	0.87	*NA*	*NA*

### Knew, shared, collaborated networks

**Fig. 2:**
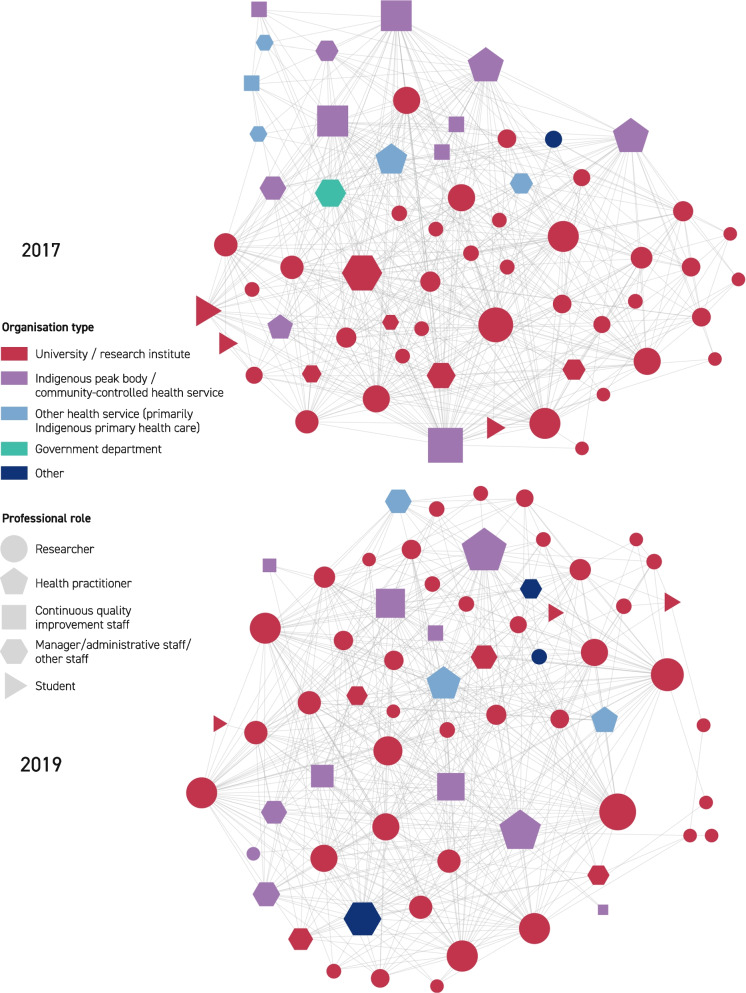
Knew before network, 2017, 2019

**Fig. 3:**
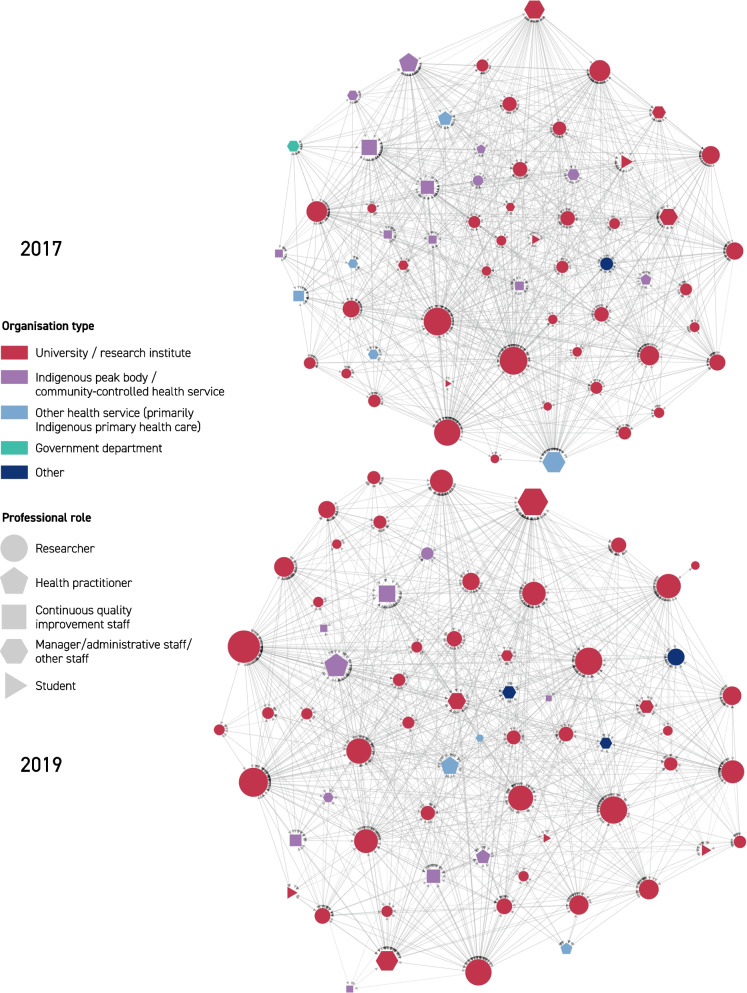
Shared information network, 2017, 2019

**Fig. 4:**
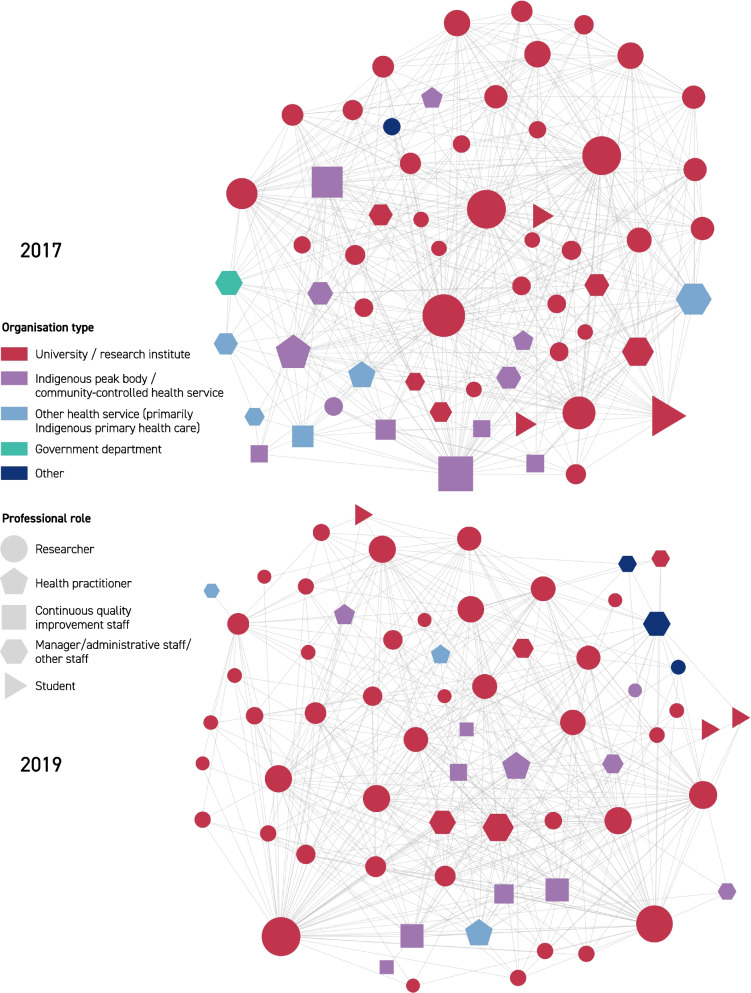
Collaborated network, 2017, 2019

Visualizations for the three networks in 2017 and 2019 are provided in Fig. 2 (knew before), Fig. 3 (shared information), and Fig. 4 (collaborated). The edges in these network diagrams reflect the responses to the social network relationship items within the surveys. The edges in Fig. 2 show which members reported knowing one another prior to the establishment of the CRE-IQI. Those edges are undirected (per network metric data in Table 2): that is, prior knowledge (of the other individual) was considered mutual for the purpose of this study, as agreed by the CRE-IQI evaluation group. In all figures, the node size represents the number of edges associated with that node (the “degree” of that node). Edges in Fig. 3 display the flow of information shared between members. As shown by the arrowheads, these edges are directed: a person may provide information to another without reciprocity. The undirected edges in Fig. 4 show the presence of collaborations.

Degree distributions for each network (the degree of a node in a network is the number of connections it has to other nodes, and the degree distribution is the probability distribution of these degrees over the whole network) showed that, although long-tailed and positively skewed, there was a characteristic scale that could be represented adequately by mean and median. The degree distribution graphs for each network in 2017 and 2019 are displayed in Additional file 3: Figs. S1, S2, S3. Although the difference in numbers is small, on average, respondents in 2017 knew more CRE-IQI members prior to the CRE-IQI, shared information with more of them and collaborated with more of them than in 2019 (17 vs 16; 20 vs 18; and 15 vs 14).

The examination of centralization (Additional file 3: Table S4) showed that all networks were moderately centralized except for the collaboration network in 2017, which had high centralization (0.77). The prior knowledge and collaboration networks were more centralized in 2017 than in 2019, indicating less reliance on nodes of high degree (i.e. influential actors) for new entrants and collaboration as the network matured. Centralization in the information-sharing networks remained the same.

The analysis of “community detection” is shown in Additional file 3: Table S5. Associations between community partition and both organization type and work role were found for the collaboration network in 2017, the prior knowledge network in 2019 and the information-sharing network in 2019. Moderate effect sizes (Cramér’s V) were observed. Between three and 17 communities were detected across networks. The moderate effect sizes computed suggest that the context of organization and role influenced the relationships in these networks.

To assess the success of the networks as an innovation platform, edge information for both surveys was analysed via three contingency table analyses using the chi-square test of independence (Table 3) covering the pairwise combinations of prior knowledge, information-sharing and collaboration.

**Table 3:** Contingency tables: (a) prior knowledge and information-sharing edges, (b) prior knowledge and collaboration edges, (c) shared information and collaboration edges, 2017 and 2019

(a) Contingency table, prior knowledge and information-sharing edges, 2017 and 2019							
		Shared information					
		2017**			2019**		
		Yes	No	Total	Yes	No	Total
Knew previously	Yes	651 (17.8%)	375 (10.2%)	1026 (28%)	319 (15.3%)	190 (9.1%)	509 (24.5%)
	No	590 (16.1%)	2044 (55.8%)	2634 (72%)	357 (17.2%)	1214 (58.4%)	1571 (75.5%)
	Total	1241 (33.9%)	2419 (66.1%)	3660 (100%)	676 (32.5%)	1404 (67.5%)	2080 (100%)

(b) Contingency table, prior knowledge and collaboration edges, 2017 and 2019							
		Collaborated					
		2017**			2019**		
		Yes	No	Total	Yes	No	Total
Knew previously	Yes	292 (16%)	221 (12.1%)	513 (28%)	255 (12.3%)	254 (12.2%)	509 (24.5%)
	No	169 (9.2%)	1148 (62.7%)	1317 (72%)	192 (9.2%)	1379 (66.3%)	1571 (75.5%)
	Total	461 (25.2%)	1369 (74.8%)	1830 (100%)	447 (21.5%)	1633 (78.5%)	2080 (100%)

(c) Contingency table, information-sharing and collaboration edges							
		Collaborated					
		2017**			2019**		
		Yes	No	Total	Yes	No	Total
Shared information	Yes	798 (21.8%)	443 (12.1%)	1241 (33.9%)	391 (18.8%)	285 (13.7%)	676 (32.5%)
	No	124 (3.4%)	2295 (62.7%)	2419 (66.1%)	56 (2.7%)	1348 (64.8%)	1404 (67.5%)
	Total	922 (25.2%)	2738 (74.8%)	3660 (100%)	447 (21.5%)	1633 (78.5%)	2080 (100%)

**Significant at the 0.001 level

*Prior knowledge and sharing of information* There was a statistically significant relationship between sharing and prior knowledge for both surveys (2017: *χ*^2^(1) = 555.24, *p* < 0.001; 2019: *χ*^2^(1) = 277.84, *p* < 0.001) (Table 3). People who knew each other previously were more likely to share information (“exploit shares”) than people with no prior relationship (“new shares”), but the proportion of “new shares” was close to half of all shares in both years. In 2017, 48% of the reported sharing (590/1241 edges) and in 2019, 53% of the reported sharing (357/676) occurred between people with no prior knowledge of each other—an indication of innovation platform success. However, people who did not know each other previously made up 85% of those who did not share information in 2017 (2044/2419 edges), and 87% in 2019 (1214/1404).

*Prior knowledge and collaboration* There was a statistically significant relationship between prior knowledge and collaboration for both surveys (2017: *χ*^2^(1) = 380.79, *p* < 0.001; 2019: *χ*^2^(1) = 324.65, p < 0.001), indicating greater collaboration between people with prior knowledge of each other (Table 3). New collaborations that occurred between previously unknown members were considered indicative of the success of the innovation platform and accounted for approximately 37% of all reported collaboration in 2017 (169/461 edges), and 43% in 2019 (192/447). However, a high proportion of people who did not know each other previously did not collaborate: 87% in 2017 (1148/1317 edges) and 88% in 2019 (1379/1571).

*Sharing information and collaboration* There was a statistically significant relationship between the sharing of information and collaboration for both surveys (2017: *χ*^2^(1) = 1524.15, *p* < 0.001; 2019: *χ*^2^(1) = 781.11, *p* < 0.001) (Table 3). Network members were more likely to share information within the collaborations than outside, with approximately 87% of collaborations in 2017 (798/922 edges) and 2019 (391/447) involving sharing of information. Approximately 36% of sharing in 2017 (443/1241 edges) and 42% of sharing in 2019 (285/676) occurred outside of direct collaborations, indicating a rising level of network support. Analysis of network paths for both the 2017 and 2019 networks showed a high likelihood of information being successfully passed between collaborators indirectly, with multiple actors in a position to have shared specific information between two or more pairs of actors. However, a high proportion of the people who did not share information did not collaborate: 95% in 2017 (2295/2419 edges) and 96% in 2019(1348/1404).

### Strategies for improving CRE-IQI effectiveness

Following Survey 1, the CRE-IQI developed strategies to address the areas suggested for improvement. Biannual meetings were held in different locations to assist with the attendance of local Indigenous health services. Indigenous co-leadership was actively encouraged in all CRE-IQI work. The online CRE-IQI newsletter adopted a new format and content to increase the promotion of the CRE-IQI. In 2019, members acknowledged improvements effected by the CRE-IQI since Survey 1 and offered suggestions for further improving the effectiveness of the CRE-IQI, including broadening its representation, involving more health services in championing the cause of CQI and the CRE-IQI, and having a greater social media presence.

## Discussion

The discussion explores findings from the network evaluation of the CRE-IQI in relation to key criteria for successful innovation platforms set out in the conceptual framework above. These criteria include the governance and management of the CRE-IQI, and the structure, conduct and performance of the CRE-IQI as an innovation platform.

### Governance and management

As an innovation platform, the CRE-IQI applied a network form of governance, with the network providing a mechanism for coordination across network members belonging to a range of different organizations. Although networks are not usually legal entities with a legal requirement for governance, such as by company boards, governance is still necessary for goal-directed organizational networks. In network forms, governance involves the use of institutions and structures of authority and collaboration to allocate resources and coordinate and control joint action across the network as a whole [31]. Hence, the role of management is critical for effective network governance [31]. In a meta-analysis of mature innovation platforms, Schut et al. found that robust organization of innovation platforms strengthened their legitimacy and sustainable impact [42].

In both surveys, members perceived that the CRE-IQI functioned well from a management perspective. Members rated it very highly for having clear leadership and meetings that were well organized and efficient. Members recognized the CRE-IQI as having a clear purpose and direction, and they perceived that participants understood and were committed to CQI and had shared goals. As noted by Swaans et al., the identification of shared goals and interests reflects a key innovation platform element [57]. Research by Scut et al. revealed that institutional embedding is associated with whether the innovation platform can strengthen systems capacity to innovate that can lead to real paradigm change [58]. Hence, it was important that members identified that their workplaces supported their involvement in the CRE-IQI. Consistent with guidance on the conduct of research with Indigenous Australians [59], there was agreement that Indigenous people co-led and directed the CRE-IQI research. However, this item was included only in the 2019 survey, and had a relatively low rating compared with other items.

### Structure of the innovation platform

According to Cadilhon’s framework for evaluating innovation platforms, aspects of structure include internal organization (including formation of a secretariat), composition and diversity of membership, and decision-making processes [39]. At CRE-IQI start-up, the coordinating centre was established with operational responsibility for the conduct of the innovation platform. As identified by Swaans et al. and supported in research by Schut et al., innovation platforms require “a facilitator who can convene and stimulate joint action” [42, 57]. Reflecting principles of operation of innovation platforms [60], members had a common vision to support joint action. A set of guiding principles on ethical research was co-developed by members and implemented to provide guidance for the CRE-IQI [49].

As an open innovation platform, the CRE-IQI was inclusive, openly inviting the inclusion of new members, consistent with innovation platform success [10, 61]. Members perceived it to be widely inclusive in the range of professional backgrounds of people involved and in actively supporting Indigenous participation. The CRE-IQI adopted an All Teach, All Learn philosophy which emphasized and valued reciprocal learning [62]. Over its life cycle, most members worked in a university or research organization and had a research role. However, the diversity of membership was reflected in representation from Indigenous community-controlled health services, Indigenous community-controlled sector support organizations, Indigenous government-operated health services, other nongovernmental organizations and a primary health network. Members also had a range of professional roles and backgrounds. Members viewed the CRE-IQI as following participatory processes (as reflected in “Membership and involvement in the CRE-IQI”, in Table S3, Additional file 3), and having dispersed leadership rather than employing top-down decision-making (as reflected in the low rating of the sub-item, “the CRE-IQI is hierarchically managed [top-down decision-making], in the item “How the CRE-IQI functions”, in Additional file 3: Table S3). These structural characteristics of the CRE-IQI are consistent with key elements of a successful innovation platform [57].

### Conduct of the innovation platform

Following Cadilhon’s framework, our analysis of innovation platform conduct included examination of information-sharing; communication; cooperation, coordination and joint planning; and trust [39]. A well-functioning and effective innovation platform should have efficient and effective knowledge and information-sharing [10, 41, 57]. The network analysis of information-sharing showed that members who knew each other prior to the CRE-IQI were more likely to share information than members with no prior relationship. Addressing information-sharing between people with no prior relationship could be an area for improvement in the CRE-IQI. On average, respondents in 2017 shared information with more members than in 2019. This may reflect a higher level of information-sharing to initiate new projects during start-up activities or to complete legacy projects predating the CRE-IQI. It is also consistent with the introduction of new “nonacademic” members in the latter years of the CRE-IQI, as it takes time to build trust and effective sharing of information and knowledge across the different sectors.

The network analyses for both 2017 and 2019 showed good “small-world” properties of the innovation platform for relaying information between members. A small-world network has a short average path length (< 2 for the CRE-IQI networks) and a high clustering coefficient (average of 0.69 for the CRE-IQI networks, where 0 is no clustering and 1 is maximal clustering) [25]. Such networks reflect the popularly known concept of “six degrees of separation” [63]. They consist of highly connected cliques or clumps linked by relatively short paths, hence facilitating the exchange of information in the networks. These findings support earlier findings from a separate co-authorship network analysis of the longer-term research collaboration from 2002 to 2019 [7]. The co-authorship network analysis showed a well-connected network in which organizations were connected by no more than two other organizations, resulting in the network being unlikely to fragment and able to disseminate information quickly [7]. The strength of the network was that it was not connected via a single dominant central organization, but rather by a core–periphery structure that pointed to a more collaborative network.

Another requirement for a well-functioning and effective innovation platform is having efficient and effective communication [57]. Homann-Kee Tui et al. identified communication as critical for facilitating the process of innovation within innovation platforms [10]. This is because the goal is to use communication processes to power changes identified by innovation platforms. Respondents perceived that the CRE-IQI had good communication with participants and that it distributed its outputs widely in the area of CQI in Indigenous PHC.

Respondents reported that the CRE-IQI facilitated interdisciplinary collaboration amongst participants. The latter is consistent with criteria for successful innovation platforms identified in research [57, 60]. Reflecting the importance of time for the development of research collaboratives [64], the network analysis revealed a higher level of collaboration between people who knew each other prior to the CRE-IQI than between people who did not. Although, on average, respondents in 2017 collaborated with more members than in 2019, the network analysis detected a high likelihood of information being passed between collaborators indirectly, as multiple actors were in a position to have shared specific information between two or more pairs. Indeed, the analysis revealed sharing of information outside of direct collaborations.

Cadilhon identified trust as an important prerequisite in innovation platforms for stakeholders to work together to solve problems [39]. The CRE-IQI had high levels of trust. Members reported that they had considerable respect for the other people involved in the CRE-IQI and confirmed that they had been able to trust participants from outside their organization to effectively contribute to project goals. Members also reported that people involved in the CRE-IQI trusted each other. These findings are consistent with findings from other CRE-IQI research, which demonstrated that the co-leadership arrangements established between Indigenous and non-Indigenous researchers on many of its projects helped create trust [49]. In addition, a network evaluation of the research collaboration predecessor to the CRE-IQI found high levels of trust across the network as a whole [6].

Members rated the face-to-face biannual meetings most highly with respect to mechanisms that were effective in developing relationships between CRE-IQI participants. According to Swaans et al., successful innovation platforms have the capacity to create spaces for long-term learning processes, particularly through iterative action–reflection learning cycles that support innovation [57]. The CRE-IQI biannual meetings served this purpose. Other mechanisms included direct introduction by another member in the usual course of their project work, and participation in masterclasses. Members reported that the CRE-IQI publications and reports were useful for their work roles, although they did not rate them as highly as they did other items.

### Performance of the innovation platform

Following Cadilhon’s framework, the performance of an innovation platform is measured according to indicators relevant to the objectives set by stakeholders at its inception [39]. Members’ mean ratings of all areas of the CRE-IQI’s functioning and achievement were higher in 2019 than in 2017—this may reflect the study design, which allowed members to address Survey 1 findings through a workshop and develop strategies for improvement. In terms of the CRE-IQI’s level of achievement in meeting its goals, members rated it most highly in both surveys for facilitating collaboration, followed by monitoring and evaluating the impact of the CRE-IQI, building CQI capacity in the Indigenous workforce, and improving the use of CQI data in clinical governance, management and practice. Members perceived that their time and effort spent with the CRE-IQI was worthwhile and that they had built new formal and informal relationships beneficial to their work. Consistent with previous innovation platform research highlighting the importance of capacity- and capability-building to support innovation across stakeholder groups [11, 42], members reported acquiring new knowledge and skills through the CRE-IQI.

In both surveys, nearly all respondents reported that their involvement in the innovation platform had assisted them in their work or their health service.

Respondents to both surveys most frequently identified the building of linkages/networking/collaboration as the most significant change effect of the CRE for them at the personal, team/work group and Indigenous PHC service level. The potential for innovation platforms to enhance communication and collaboration of stakeholders to stimulate innovation was recognized in previous research [57, 60, 61]. For both surveys, the next most significant change effect of the CRE for respondents was capacity-building, including knowledge transfer and skills in CQI, and career development/mentoring. Previous research identified capacity-building as important to sustaining innovation platforms [11, 42]. At the wider system level, in 2017, research translation was seen as the most significant change, while in 2019, the capacity-building skills of knowledge transfer and skills in CQI were mentioned most frequently. The latter finding is supported in previous CRE-IQI research [7], providing further support that the capacity-building achievements of the CRE-IQI were aligned with requirements identified in research on successful innovation platforms [11, 22, 42]. Reflecting the impact of the CRE-IQI at the system level in 2019, members next identified policy impact as the most significant change. Homann-Kee Tui et al. also noted that innovation platforms could have an important role in influencing policy-makers at the optimal time [10]. The provision of research evidence on CQI was identified by members as the next most significant change at the system level in 2019. Consistent with best practice operating principles of innovation platforms [10], the CRE-IQI provided the opportunity for stakeholder identification of research priorities in CQI research, resulting in research evidence that was relevant to stakeholders.

This study addresses a gap in the literature, as it examines an innovation platform at different system levels [22]. While innovations require long-term changes that can be difficult to assess in the shorter term [22], a number of intermediate outcomes at different system levels reflected early stage changes for this innovation platform that could be documented with empirical evidence. Other CRE-IQI research also identified the importance of having sufficient time to see innovations through and to establish the social system enabling interconnectivity between members [48].

### Study strengths and limitations

A strength of the network evaluation was the opportunity to examine the innovation platform over its life cycle through data collection at two successive time points. Use of network metrics allowed for the objective measurement of information-sharing and collaboration in the CRE-IQI. Analysis of midpoint data provided the opportunity to review data and develop improvement strategies. As the study was part of a wider evaluation of the CRE-IQI, findings were applied in conjunction with other evaluation approaches to strengthen the overall evaluation of the innovation platform.

It is important to note some possible limitations of this study. Although the evaluation was guided by the domains identified in the literature on innovation platform evaluation, these domains might not cover all the relevant functions of a well-functioning innovation platform. In the evaluation time frame, only evidence of intermediate outcomes for the innovation platform could be identified—not long-term innovations. Further, although the CRE-IQI evolved continuously over its life cycle, the analysis reflects the network observed at two time points (including retrospective data collection on network relationships at commencement). In addition, for collection of data on the initial network, respondents were asked later in the network’s life cycle to identify whom they knew at the start of the CRE-IQI, from a bounded list, and recall might not have been complete. Survey 2 used an additional snowballing step in sampling, and the difference in the survey methods for each survey means that comparisons between surveys are subject to this limitation. However, the difference in methods can be justified—the network had developed and grown, and simply repeating the exact methods from Survey 1 would have compromised our ability to capture some of these changes. For the perception data, the positive feedback may reflect so-called survivorship bias [65], because respondents continued to be involved in the CRE-IQI, whereas the views of those who left could have been less favourable. However, innovation platforms do evolve, and those who no longer align with changes and new directions naturally leave to make room for “new blood” who align with the new directions and bring fresh ideas.

## Conclusion

The network evaluation shows that the CRE-IQI was successful as an innovation platform in creating a multi-stakeholder collaboration that built the CQI capacity of researchers and services and produced research evidence on CQI to improve health outcomes in Indigenous PHC settings and impact on health policy. The innovation platform had good “small-world” network properties for relaying information in both information-sharing and collaboration networks. This study demonstrates the application of network analysis in assessing the innovation platform’s network at different time points in its life cycle, thereby providing the opportunity for midpoint findings to inform the development and implementation of strategies to improve its functioning. This research adds to our understanding of enabling factors for successful innovation platforms and will inform the further application of innovation platforms in the health sector in Australia and internationally.

## Supplementary Information

**Additional file 1.** CRE-IQI network survey, 2017.

**Additional file 2.** CRE-IQI network survey, 2019.

**Additional file 3: Table S1.** Definition of social network terms; **Table S2.** Most significant change made by the CRE-IQI for respondents at four levels, 2017 and 2019; **Table S3.** Members’ overall perceptions of CRE-IQI, 2017 and 2019; **Table S4.** Degree centralization of networks, 2017 and 2019; **Table S5.** Community detection in networks, 2017 and 2019; **Figure S1.** Degree distribution for network of prior knowledge, 2017 and 2019; **Figure S2.** Degree distribution for network of information-sharing, 2017 and 2019; **Figure S3.** Degree distribution for network of collaborations, 2017 and 2019.

## Data Availability

Consistent with ethics approval requirements for this study, the datasets for this study are not publicly available, as they contain personal relationship data.

## Abbreviations

CQI: Continuous quality improvement
CRE: Centre for Research Excellence
CRE-IQI: Centre for Research Excellence in Integrated Quality Improvement
IQI: Integrated quality improvement
PHC: Primary healthcare
SNA: Social network analysis
