# Supplementary material for: Network evaluation of an innovation platform in continuous quality improvement in Australian Indigenous primary healthcare
Source: Health Res Policy Syst. 2022 Oct 31;20:119. doi: 10.1186/s12961-022-00909-z (PMC9620635; doi:10.1186/s12961-022-00909-z)
Supplement: Supplementary file 1 — Additional file 1. CRE-IQI network survey, 2017. [file 12961_2022_909_MOESM1_ESM.pdf]

CRE-IQI Network Survey

## Welcome to the CRE-IQI Network Survey

**Welcome to the Network Survey for the Centre for Research Excellence in Integrated Quality Improvement.**

**Thank you for taking part in this important survey to help in evaluating the CRE-IQI. Today we will be gaining your thoughts and opinions to assess how the CRE is functioning as a research collaboration and as an Innovation Platform. Your feedback will help us to find out what is working well, and where there may be a need to address any issues. All answers you provide will be kept in the strictest confidentiality. Upon receipt of your completed survey, the responses will be coded and de-identified. We will report findings to participants through the CRE-IQI.**

**The survey will take around 20-25 minutes to complete.**

* 1. By clicking 'Yes' here, you consent to take part in this survey. Your responses will remain confidential.

- ◯ Yes, I agree to take part in this survey
- ◯ No, I do not want to take part in this survey

CRE-IQI Network Survey

## A. Background Information

**Instructions**

Responses from all CRE-IQI network participants will help to ensure the reliability of research on the whole network. Please read through carefully and answer every question. Thoughtful, honest responses will help the CRE to learn about its strengths and weaknesses, and to improve its effectiveness.

* 2. Please select which of the following best describes the organisation where you work primarily, or your affiliation:

- Aboriginal & Torres Strait Islander Community Controlled Health Organisation
- Community controlled peak body
- Government operated health service
- University or research organisation
- Other (please specify)
- Government department
- Other government organisation
- Other non-government organisation
- Primary Health Network

* 3. Please state the postcode of your employment:

* 4. Please indicate your gender

- Female
- Male
- Other

* 5. What is your age group?

- Less than 25 years
- 25 to 39 years
- More than 40 years

CRE-IQI Network Survey

## A. Background Information

* 6. What is your primary work position?

- ◯ Aboriginal and/or Torres Strait Islander Health Practitioner/Worker
- ◯ Public Health Physician
- ◯ Other Medical Practitioner
- ◯ Director/Board Member
- ◯ Quality Improvement Facilitator/Coordinator
- ◯ Allied Health Professional
- ◯ Policy Officer
- ◯ Project Officer
- ◯ Other (please specify)
- ◯ Researcher/Academic
- ◯ Nurse/Midwife
- ◯ Health Centre Manager
- ◯ Senior Manager/Executive
- ◯ Manager
- ◯ Health Promotion Officer
- ◯ PhD Student

* 7. How long have you been working in your present position?

- ◯ Less than 6 months
- ◯ 6 months - 2 years
- ◯ 2 - 5 years
- ◯ More than 5 years

* 8. What is your professional background?

- ◯ Medical Practitioner
- ◯ Aboriginal and/or Torres Strait Islander Health Practitioner/Worker
- ◯ Manager or Administrator
- ◯ Researcher
- ◯ Director (e.g. Board Director)
- ◯ Allied Health Professional
- ◯ Other (please specify)
- ◯ Registered Nurse
- ◯ Enrolled Nurse
- ◯ Quality Improvement Facilitator
- ◯ Health Promotion Officer
- ◯ Policy or Planning Officer

* 9. How many years experience have you had in your profession?

- Less than 5 years
- 5 - 9 years
- 10 - 19 years
- More than 20 years

* 10. Do you have expertise in a particular area relating to continuous quality improvement (CQI) in Indigenous primary health care?

- Yes
- No

## A. Background Information

11. My area of expertise is:

[ ]

* 12. Do you identify as being of Aboriginal and/or Torres Strait Islander origin?

- ◯ Yes, Aboriginal
- ◯ Yes, Torres Strait Islander
- ◯ Yes, both
- ◯ No

* 13. Were you involved with the previous ABCD program of work (such as the ABCD National Research Partnership)?

- ◯ Yes
- ◯ No
- ◯ I do not know

* 14. How long have you been involved with the CRE-IQI? (The CRE was established in December 2014)

- ◯ Less than 6 months
- ◯ 6 months - 1 year
- ◯ 1 - 2 years
- ◯ More than 2 years

## A. Background Information

* 15. How many CRE-IQI meetings did you attend in the last 12 months?

| | 0 | 1 | 2 | 3 | 4 | 5-6 | 7-8 |
|---|---|---|---|---|---|---|---|
| CRE Face to Face Biannual Meetings (up to 2 meetings) | ◯ | ◯ | ◯ | ◯ | ◯ | ◯ | ◯ |
| Management Committee Meetings (up to 6 meetings) | ◯ | ◯ | ◯ | ◯ | ◯ | ◯ | ◯ |
| Research Capacity Building Teleconferences (up to 8 meetings) | ◯ | ◯ | ◯ | ◯ | ◯ | ◯ | ◯ |
| CRE Masterclasses (up to 4 meetings) | ◯ | ◯ | ◯ | ◯ | ◯ | ◯ | ◯ |
| CRE Face to Face Evaluation Team Meetings (up to 2 meetings) | ◯ | ◯ | ◯ | ◯ | ◯ | ◯ | ◯ |

* 16. Have the following CRE-IQI materials been useful for you in your work?

| | Not at all useful | Moderately useful | Very useful | Extremely useful |
|---|---|---|---|---|
| E-Newsletters | ◯ | ◯ | ◯ | ◯ |
| Website | ◯ | ◯ | ◯ | ◯ |
| Facebook page | ◯ | ◯ | ◯ | ◯ |
| Reports | ◯ | ◯ | ◯ | ◯ |
| Peer-reviewed publications | ◯ | ◯ | ◯ | ◯ |

* 17. What is the major objective **you** hope to accomplish through the CRE-IQI with regard to improving the health outcomes of Indigenous Australians?

- To increase understanding of, or identify, priority areas in CQI in Indigenous primary health care
- To build relationships, create a coalition of interest or support for priority issues in CQI in Indigenous primary health care
- To develop and progress research relating to CQI in Indigenous primary health care
- Other (please specify)
- To develop and progress research methods and techniques
- To translate research and evidence into practice

## A. Background Information

* 18. With which of these CRE-IQI Flagship projects have you been involved?

- [ ] Engaging Stakeholders in Identifying Priority Evidence-Practice Gaps & Strategies for Improvement (ESP) Project (Investigator: Prof. Ross Bailie)
- [ ] Lessons from the Best (Investigator: Prof. Sarah Larkins)
- [ ] Ongoing ABCD Data Analysis (Investigator: Prof. Ross Bailie)
- [ ] CQI and Maternal Health Project (Investigator: Dr. Melanie Gibson-Helm)
- [ ] CQI and Social and Emotional Well-being Project (Investigator: Prof. Komla Tsey)
- [ ] Monitoring and Evaluation of the CRE-IQI as an Innovation Platform
- [ ] None of these

* 19. With which CRE-IQI Research or Work Program(s) have you been involved, or are you currently involved?

- [ ] Aim 1: Refining and building new processes and tools
- [ ] Aim 2: Improving data reporting systems
- [ ] Aim 3: Improving use of QI data in clinical governance, management and practice
- [ ] Aim 4: Building QI capacity in the Indigenous workforce
- [ ] Aim 5: Monitoring and evaluating the impact of the CRE/Innovation Platform
- [ ] Work Program 1: Promoting transfer of research outcomes into health policy/practice
- [ ] Work Program 2: Developing the capacity of the health and medical research workforce
- [ ] Work Program 3: Facilitating collaboration

* 20. Which aspect(s) best describes **your** contribution to the goals of the CRE-IQI?

- [ ] Working on collaborative and applied research in CQI on Indigenous primary health care
- [ ] Development of tools and methods for CQI in Indigenous primary health care
- [ ] Applying evaluation methods relating to the CRE and its projects
- [ ] Providing service level input into the work of the CRE
- [ ] Applying or disseminating research evidence on CQI
- [ ] Building capacity of the research workforce in CQI in Indigenous primary health care
- [ ] Developing evidence-based policies and guidelines
- [ ] Other (please specify)

* 21. Has your involvement in the CRE-IQI assisted you in your work, or in your health service?

- ( ) Yes
- ( ) No

## A. Background Information

22. Please give an example (or examples) of how your involvement in the CRE-IQI has assisted you in your work, or in your health service:

## B. Your Experience of the CRE-IQI as an Innovation Platform

* 23. How would you assess the CRE-IQI's level of achievement in meeting each of these goals?

| | Very poor | Poor | Good | Very good |
|---|---|---|---|---|
| Refining and building new processes and tools | ◯ | ◯ | ◯ | ◯ |
| Improving data reporting systems | ◯ | ◯ | ◯ | ◯ |
| Improving use of QI data in clinical governance, management and practice | ◯ | ◯ | ◯ | ◯ |
| Building QI capacity in the Indigenous workforce | ◯ | ◯ | ◯ | ◯ |
| Monitoring and evaluating impact of the CRE | ◯ | ◯ | ◯ | ◯ |
| Promoting transfer of research outcomes into health policy/practice | ◯ | ◯ | ◯ | ◯ |
| Developing the capacity of the health and medical research workforce | ◯ | ◯ | ◯ | ◯ |
| Facilitating collaboration | ◯ | ◯ | ◯ | ◯ |

* 24. To what extent do you agree or disagree with each of these statements about**how the CRE-IQI works** most of the time?

| | Strongly disagree | Disagree | Agree | Strongly agree |
|---|---|---|---|---|
| The CRE has a clear purpose and direction | ◯ | ◯ | ◯ | ◯ |
| CRE participants understand the CRE goals | ◯ | ◯ | ◯ | ◯ |
| The CRE has a realistic timetable for delivery of its work program | ◯ | ◯ | ◯ | ◯ |
| CRE members understand and are committed to CQI | ◯ | ◯ | ◯ | ◯ |
| There is clear leadership of (or champions for) the CRE | ◯ | ◯ | ◯ | ◯ |
| Only a few are involved in discussion, not everyone | ◯ | ◯ | ◯ | ◯ |
| The CRE Coordinating Centre is critical to the CRE effectiveness | ◯ | ◯ | ◯ | ◯ |
| CRE meetings are organised and efficient | ◯ | ◯ | ◯ | ◯ |
| The CRE is hierarchically managed (top-down decision-making) | ◯ | ◯ | ◯ | ◯ |
| My workplace is supportive of my involvement in the CRE | ◯ | ◯ | ◯ | ◯ |

## B. Your Experience of the CRE-IQI as an Innovation Platform

* 25. To what extent do you agree or disagree with each of these statements about **membership and involvement** in the CRE-IQI?

| | Strongly disagree | Disagree | Agree | Strongly agree |
|---|---|---|---|---|
| People involved in the CRE trust each other | | | | |
| The CRE uses the abilities of just a few, not all | | | | |
| The CRE has the right people to achieve CRE outcomes | | | | |
| The CRE is widely inclusive in the range of professional backgrounds of people involved | | | | |
| The CRE is widely inclusive of people with different levels of professional seniority | | | | |
| The CRE actively supports Indigenous participation | | | | |
| The CRE participants include expertise in CQI in Indigenous primary healthcare practice | | | | |
| The CRE participants include expertise in research on CQI in Indigenous primary health care | | | | |
| The CRE actively involves community-controlled Indigenous health services | | | | |
| The CRE actively involves government-operated Indigenous health services | | | | |

| | Strongly disagree | Disagree | Agree | Strongly agree |
|---|---|---|---|---|
| The CRE actively involves practising health care practitioners | | | | |
| The CRE actively involves CQI facilitators and coordinators | | | | |
| The CRE actively involves health service managers | | | | |
| The CRE actively involves policy makers | | | | |

## B. Your Experience of the CRE-IQI as an Innovation Platform

* 26. To what extent do you agree or disgree with each of these following statements about **communication** in the CRE-IQI?

| | Strongly disagree | Disagree | Agree | Strongly agree |
|---|---|---|---|---|
| The CRE has good communication and coordination with CRE participants | ○ | ○ | ○ | ○ |
| The CRE has facilitated inter-disciplinary collaboration amongst CRE participants | ○ | ○ | ○ | ○ |
| The CRE has not improved information sharing between CRE participants | ○ | ○ | ○ | ○ |
| The CRE disseminates its outputs widely in the area of CQI in Indigenous primary health care | ○ | ○ | ○ | ○ |

* 27. To what extent do you agree or disagree with each of these statements in relation to CRE-IQI **effects or impacts?**

| | Strongly disagree | Disagree | Agree | Strongly agree |
|---|---|---|---|---|
| I have acquired new knowledge and skills through the CRE | ○ | ○ | ○ | ○ |
| I have built new formal relationships beneficial to my work | ○ | ○ | ○ | ○ |
| I have built new informal relationships beneficial to my work | ○ | ○ | ○ | ○ |
| The CRE has facilitated the effective use of data for quality improvement | ○ | ○ | ○ | ○ |
| The CRE has facilitated acquisition of additional funding or other resources | ○ | ○ | ○ | ○ |
| CRE collaboration has resulted in new research in CQI in Indigenous primary health care | ○ | ○ | ○ | ○ |
| My time and effort spent with the CRE is worthwhile | ○ | ○ | ○ | ○ |

## B. Your Experience of the CRE-IQI as an Innovation Platform

* 28. In the last 12 months, to what extent has each of these been a barrier or a facilitator to the CRE-IQI's ability to improve CQI in Indigenous primary health care?

| | Major barrier | Minor barrier | Minor facilitator | Major facilitator |
|---|---|---|---|---|
| Financial resources | ◯ | ◯ | ◯ | ◯ |
| Human resources (e.g., staff, professional expertise) | ◯ | ◯ | ◯ | ◯ |
| Physical resources (e.g., sites for meetings, education) | ◯ | ◯ | ◯ | ◯ |
| Existing regulations and policies | ◯ | ◯ | ◯ | ◯ |
| Infrastructure to collect and analyse data | ◯ | ◯ | ◯ | ◯ |
| Attitudes and beliefs held by health professionals and organisations | ◯ | ◯ | ◯ | ◯ |

* 29. Can you please state the most significant change that the CRE has made at each of these levels:

For yourself

For your team/work group

For Indigenous primary health care services

For the wider system leve

* 30. The CRE-IQI was established in December 2014 as an innovation Platform to foster collaborations for priority-driven research and to strengthen CQI efforts in Indigenous primary health care. Overall, do you think that the CRE is achieving its purpose?

- ◯ Not at all
- ◯ To a small extent
- ◯ To some extent
- ◯ To a considerable extent
- ◯ To a great extent

## C. Collaboration Information

It is very important that you answer the question below for EACH person that you **(1)** knew before your initial participation in the CRE-IQI (December 2014), or **(2)** gave information to, or **(3)** received information from, or **(4)** have collaborated with on research or a project. Note that some of the Questions are similar. Please read each column-question carefully and answer it independently of any other column question.

* 31. Firstly, whom did you:
**(1) Know before you participated** in the CRE-IQI (i.e. professionally or socially)
Secondly, in the last 6 months (outside of formal CRE meetings e.g. Biannual Meetings), please select anyone on the list whom:
**(2) You have given information** or **advice** to relating to the CRE and its work, and whom
**(3) You have received information** or **advice** from relating to the CRE and its work, and whom
**(4) You have collaborated with** on CRE related research or a CRE related project;
Or,
**(5) Select 'Not Applicable or (6) 'Self'**

Please tick ALL applicable boxes in each row.

| | (1) Knew before the CRE | (2) Given information to | (3) Received information from | (4) Collaborated with | (5) Not Applicable | (6) Self |
|---|---|---|---|---|---|---|
| *e.g.JohnSmith*, Employer Organisation... | ☐ | ☐ | ☐ | ☐ | ☐ | ☐ |
| [CRE-IQI member names/details deleted for privacy reasons] | ☐ | ☐ | ☐ | ☐ | ☐ | ☐ |
| …………., ……………...... …………….. | ☐ | ☐ | ☐ | ☐ | ☐ | ☐ |
| ………………..,….. …………… ……………. | ☐ | ☐ | ☐ | ☐ | ☐ | ☐ |
| ……………. ……………. | ☐ | ☐ | ☐ | ☐ | ☐ | ☐ |
| ………… ... .. …………………e ……… | ☐ | ☐ | ☐ | ☐ | ☐ | ☐ |
| ……… ……... ……………….. | ☐ | ☐ | ☐ | ☐ | ☐ | ☐ |
| ……………… ………………. ……………. | ☐ | ☐ | ☐ | ☐ | ☐ | ☐ |
| …………….. ……………….. | ☐ | ☐ | ☐ | ☐ | ☐ | ☐ |

| | (1) Knew before the CRE | (2) Given information to | (3) Received information from | (4) Collaborated with | (5) Not Applicable | (6) Self |
|---|---|---|---|---|---|---|
| …………….., …………… | ☐ | ☐ | ☐ | ☐ | ☐ | ☐ |
| …………., …………… .. ………. | ☐ | ☐ | ☐ | ☐ | ☐ | ☐ |
| ………………., ……………….. ………. | ☐ | ☐ | ☐ | ☐ | ☐ | ☐ |
| …………., …………….. …………. | ☐ | ☐ | ☐ | ☐ | ☐ | ☐ |
| ………………… …………… | ☐ | ☐ | ☐ | ☐ | ☐ | ☐ |
| ………………… ………………….. ……… | ☐ | ☐ | ☐ | ☐ | ☐ | ☐ |
| …………., ………………….. …………. | ☐ | ☐ | ☐ | ☐ | ☐ | ☐ |
| ………. …………….. ……………. …………... | ☐ | ☐ | ☐ | ☐ | ☐ | ☐ |
| ……………………. ………………. ……………. | ☐ | ☐ | ☐ | ☐ | ☐ | ☐ |
| ……………………. ………………… ……………. | ☐ | ☐ | ☐ | ☐ | ☐ | ☐ |
| …………… ……………. ………………….. ………. | ☐ | ☐ | ☐ | ☐ | ☐ | ☐ |
| ……………… ……………… …………. | ☐ | ☐ | ☐ | ☐ | ☐ | ☐ |
| ……………… …………. ……………….. …………… ………………. | ☐ | ☐ | ☐ | ☐ | ☐ | ☐ |
| …………., ……………………… ………. | ☐ | ☐ | ☐ | ☐ | ☐ | ☐ |
| ……………. ……………… …………………... | ☐ | ☐ | ☐ | ☐ | ☐ | ☐ |

| | (1) Knew before the CRE | (2) Given information to | (3) Received information from | (4) Collaborated with | (5) Not Applicable | (6) Self |
|---|---|---|---|---|---|---|
| …………………<br>………………….. | ☐ | ☐ | ☐ | ☐ | ☐ | ☐ |
| …………….<br>…………………<br>……………. | ☐ | ☐ | ☐ | ☐ | ☐ | ☐ |
| ………<br>…………………..<br>……………….<br>………… | ☐ | ☐ | ☐ | ☐ | ☐ | ☐ |
| ……………<br>…………<br>………………… | ☐ | ☐ | ☐ | ☐ | ☐ | ☐ |
| …………………<br>…………….<br>…………. | ☐ | ☐ | ☐ | ☐ | ☐ | ☐ |
| ………………….<br>…………………. | ☐ | ☐ | ☐ | ☐ | ☐ | ☐ |
| ………………….<br>…………………<br>…………………… | ☐ | ☐ | ☐ | ☐ | ☐ | ☐ |
| ………………..<br>………………… | ☐ | ☐ | ☐ | ☐ | ☐ | ☐ |
| …………..<br>……………….<br>…………. | ☐ | ☐ | ☐ | ☐ | ☐ | ☐ |
| …………<br>………………..<br>………… | ☐ | ☐ | ☐ | ☐ | ☐ | ☐ |
| ……………………<br>……………………<br>……………… | ☐ | ☐ | ☐ | ☐ | ☐ | ☐ |
| ………………..<br>………………….<br>……………… | ☐ | ☐ | ☐ | ☐ | ☐ | ☐ |
| …………..<br>…………<br>………………<br>…………. | ☐ | ☐ | ☐ | ☐ | ☐ | ☐ |
| ………………<br>…………………<br>………………….<br>………………….. | ☐ | ☐ | ☐ | ☐ | ☐ | ☐ |
| ……………….<br>……………….<br>…………………<br>……………. | ☐ | ☐ | ☐ | ☐ | ☐ | ☐ |
| …………….<br>……………………….<br>….. | ☐ | ☐ | ☐ | ☐ | ☐ | ☐ |

| | (1) Knew before the CRE | (2) Given information to | (3) Received information from | (4) Collaborated with | (5) Not Applicable | (6) Self |
|---|---|---|---|---|---|---|
| ……………………..<br>………………………<br>…………… | ☐ | ☐ | ☐ | ☐ | ☐ | ☐ |
| ……....................<br>………………… | ☐ | ☐ | ☐ | ☐ | ☐ | ☐ |
| ………….<br>……………………..<br>…………. | ☐ | ☐ | ☐ | ☐ | ☐ | ☐ |
| …………….<br>………..<br>………………..<br>……………. | ☐ | ☐ | ☐ | ☐ | ☐ | ☐ |
| …………….<br>…………………<br>………………..<br>…………… | ☐ | ☐ | ☐ | ☐ | ☐ | ☐ |
| ……………….<br>……………………..<br>………………. | ☐ | ☐ | ☐ | ☐ | ☐ | ☐ |
| ……………….<br>…………………<br>…………… | ☐ | ☐ | ☐ | ☐ | ☐ | ☐ |
| ………….<br>………………. | ☐ | ☐ | ☐ | ☐ | ☐ | ☐ |
| …………<br>……………….<br>……. ……..<br>………. | ☐ | ☐ | ☐ | ☐ | ☐ | ☐ |
| …………………..<br>………<br>…………..<br>………………… | ☐ | ☐ | ☐ | ☐ | ☐ | ☐ |
| …. ………. …..<br>……………….<br>…………. | ☐ | ☐ | ☐ | ☐ | ☐ | ☐ |
| …………………<br>………………..<br>……………….. | ☐ | ☐ | ☐ | ☐ | ☐ | ☐ |
| ……………..<br>……………..<br>………………… | ☐ | ☐ | ☐ | ☐ | ☐ | ☐ |
| ……………..<br>…………………<br>……… | ☐ | ☐ | ☐ | ☐ | ☐ | ☐ |
| ……………….<br>………..<br>………………… | ☐ | ☐ | ☐ | ☐ | ☐ | ☐ |

| | (1) Knew before the CRE | (2) Given information to | (3) Received information from | (4) Collaborated with | (5) Not Applicable | (6) Self |
|---|---|---|---|---|---|---|
| …………<br>………………<br>……………….. | ☐ | ☐ | ☐ | ☐ | ☐ | ☐ |
| ……………...<br>………………. | ☐ | ☐ | ☐ | ☐ | ☐ | ☐ |
| ……………….<br>…………………. | ☐ | ☐ | ☐ | ☐ | ☐ | ☐ |
| ……………<br>……………………..<br>…………. | ☐ | ☐ | ☐ | ☐ | ☐ | ☐ |
| ………………..<br>………………….<br>……… | ☐ | ☐ | ☐ | ☐ | ☐ | ☐ |
| ………….<br>……………… …..<br>……………… | ☐ | ☐ | ☐ | ☐ | ☐ | ☐ |

* 32. In addition to the list of people above, could you please estimate the number of people you have sent information relating to the CRE? Please consider estimates of conference presentation audiences, and readership of reports and journal publications. (As a guide, this number could be between 0 and 10,000).

33. Please provide your suggestions (if any) for improving the effectiveness of the CRE-IQI as a collaborative network.

**THANK YOU**

Your time in completing the survey is very much appreciated.
