# Supplementary material for: Network evaluation of an innovation platform in continuous quality improvement in Australian Indigenous primary healthcare
Source: Health Res Policy Syst. 2022 Oct 31;20:119. doi: 10.1186/s12961-022-00909-z (PMC9620635; doi:10.1186/s12961-022-00909-z)
Supplement: Supplementary file 2 — Additional file 2. CRE-IQI network survey, 2019. [file 12961_2022_909_MOESM2_ESM.pdf]

PARTICIPANT INFORMATION SHEET

This is for you to keep

Invitation to Participate

Can you please help us by participating in the 2019 Network Survey of the Centre for Research Excellence in Integrated Quality Improvement (CRE-IQI)? The CRE-IQI has been established as an innovation platform for systems-wide improvement in continuous quality improvement in primary health care for Aboriginal and Torres Strait Islander Australians, and has now been underway since late 2014.

What will this survey involve?

This survey is part of the evaluation of the CRE-IQI as an innovation platform. The survey includes assessment of the CRE-IQI's research and work networks to assess the extent to which the CRE-IQI has facilitated collaboration across participants and between participants and external end users.

We wish to seek your feedback on your participation in the CRE-IQI through an online survey.

The survey will help us understand the CRE-IQI network structure, CRE-IQI research collaboration, and information dissemination from the CRE-IQI. If you complete the survey you will be invited to participate in a draw to win a gift voucher (see details below).

How long will it take?

The survey will take approximately 20-30 minutes to complete.

Participation

We are inviting you to complete the survey because you have participated in the CRE-IQI and its research and work programs. Your consent to participate will be requested for the survey. If you agree to participate, you can change your mind and withdraw at any time. Your decision will in no way impact upon your current or future relationship with your employer or the program partners.

Outline of the Project

The key topic areas addressed in the surveys include:

- Background Information – asks for some background information about you.
- Collaboration & Network Information – asks relationship questions regarding collaboration in the network: whom you know in the research network; who you have sent information or material to in the research network; and who you have worked with on research or other work in the research network.
- Network Experience – asks questions relating to your experience of the CRE-IQI network.

Expected Benefits

Your involvement in the research will assist with feedback on the functioning of the CRE-IQI to inform the network evaluation of the CRE-IQI.

Rewards

Participants who complete the survey go into the draw to win 1 of 10 vouchers of $50 value for Coles-Myer (or local equivalent if applicable). Draws will take place on 01 September 2019 and winners will be contacted by the research team via phone and email.

Risks

There are no foreseeable risks related to your involvement in the surveys. However, you can withdraw at any time if you feel uncomfortable. If you decide to withdraw we will not use your information.

Confidentiality

We will remove any identifying information (such as your name and job title) from the survey form, and a non-identifiable code will be given for each survey respondent. The coding list will be stored separately from your survey responses. The researchers give their assurances that all information you provide is strictly confidential to the project team and will not be given to anyone else. No individual or organisation will be identified in reports, publications or presentations. The funding body will not have access to the information provided by individuals who participate in these surveys.

All information collected in this survey will be stored electronically on the Menzies School of Health Research secure network. This will only be accessed by staff working directly on the Network Evaluation of the CRE in Integrated Quality Improvement and the data will be deleted after the minimum storage period of five years.

Questions / Concerns / Complaints regarding the conduct of the study

If you have any questions, concerns or complaints about this project please contact:
Dr Frances Cunningham, Senior Research Fellow
Division of Wellbeing and Preventable Chronic Disease
Menzies School of Health Research
Email: frances.cunningham@menzies.edu.au
Phone: (07) 3169 4219
Level 1, 147 Wharf Street, Spring Hill, QLD 4000

This project has been approved by the following Human Research Ethics Committee:
Aboriginal Ethics Sub-Committee (AESC) of the Human Research Ethics Committee of the Northern Territory Department of Health and Menzies School of Health Research HREC (Approval No. HREC-2017-2859) in accordance with the NHMRC National Statement on Ethical Conduct in Human Research.

As the lead organisation on the project, Menzies School of Health Research is committed to researcher integrity and the ethical conduct of research. Please contact the Principal Investigator (contact above) if you have any questions, concerns or require further information about the study

If you have any concerns or complaints regarding the ethical conduct of the study, you are invited to contact the Ethics Administration Officer, Human Research Ethics Committee of the Northern Territory Department of Health and Menzies School of Health Research on (08) 8946 8687 or (08) 8946 8692 or email ethics@menzies.edu.au

INFORMED CONSENT FORM

This means you can say NO

I agree that

i. I have read the information provided about the purpose of the Network Evaluation of the CRE in Integrated Quality Improvement as an Innovation Platform and the aims of the Participant Survey.

   This has included information about:

   - Who is funding the project
   - Who is involved in the project
   - What my participation in this survey involves
   - What the risks and benefits of my participation are
   - How my personal information is being stored and protected
   - Who owns the information I provide
   - How information collected by the project will be used
   - How to contact the Principal Investigator from Menzies School of Health Research to ask questions about the project
   - How to contact the Menzies School of Health Research Ethics Committee to make a complaint about the ethical conduct of the project.

ii. I have been given a Participant Information Sheet describing all of the above points.

iii. I understand all of the above points and have been able to ask questions about anything that is unclear.

iv. I agree to complete a 20-30 minute participant survey for the Network Evaluation of the CRE in Integrated Quality Improvement.

Q1.1 **By ticking 'Yes' here, you consent to take part in this survey. Your responses will remain confidential.**

○ Yes, I agree to take part in this survey

○ No, I do not want to take part in this survey

1. INTRODUCTION

Welcome to the final Network Survey for the Centre for Research Excellence in Integrated Quality Improvement (CRE-IQI).

Thank you for taking part in this important survey to help in evaluating the CRE-IQI. This follow-up on the first network survey in 2017-18 will assess how the CRE-IQI is functioning as a research collaboration and as an Innovation Platform using social network analysis.

We will remove any identifying information (such as your name and job title) from the survey form, and a non-identifiable code will be given for each survey respondent. The coding list will be stored separately from your survey responses. The researchers give their assurances that all information you provide is strictly confidential to the project team and will not be given to anyone else.

All answers you provide will be kept in the strictest confidentiality. Findings will be reported to participants through the CRE-IQI. No identifiable information will be reported.

This survey will take between 20 and 30 minutes to complete.

INSTRUCTIONS:

Please read through carefully and answer every item in the survey. Responses from all CRE-IQI participants will help ensure the reliability of research on the whole network. Your responses will help us to understand the CRE as a network, find out what has worked well and develop insights for further successful collaborations in the future.

2. BACKGROUND INFORMATION

Q2.1 **Your first name:**	
Q2.2 **Your surname:**	

Q2.3 **Please select which of the following best describes the organisation where you work (or worked) in the last 12 months primarily <u>in relation to the CRE-IQI</u>**

(You may nominate an additional place of work if it is also relevant to your time with the CRE-IQI)

	**Please mark with ✓**	
	**Primary place of work**	**Additional place of work**
Aboriginal & Torres Strait Islander Community Controlled Health Organisation		
Community controlled peak body		
Government operated health service		
University or research organisation		
Government department		
Other government organisation		
Other non-government organisation		
Primary Health Network		
Other (please specify) ________________________________		

Q2.4 **Please state the postcode of the primary place of work noted above:**

____

Q2.5 **Please indicate your gender (please circle one)**

Female	Male	Other

Q2.6 **What is your age group? (please circle one)**

Less than 25 years	25 to 39 years	More than 40

Q2.7 **Do you identify as being of Aboriginal and/or Torres Strait Islander origin? (please circle one)**

Yes, Aboriginal	Yes, Torres Strait Islander
Yes, both	No

Q2.8 **What was your primary work position in the last 12 months, <u>relating to any work done with the CRE-IQI</u>? Please select only one primary position and one secondary position if relevant**

	**Please mark with ✓**	
	**Primary work position** (select one only)	**Secondary work position** (select one if relevant)
Aboriginal and/or Torres Strait Islander Health Practitioner/Worker		
Public Health Physician		
Other Medical Practitioner		
Director/Board Member		
Quality Improvement Facilitator/Coordinator		
Allied Health Professional		
Policy Officer		
Project Officer		
Early Career Researcher		
Researcher/Academic		
Nurse/Midwife		
Health Centre Manager		
Senior Manager/Executive		
Manager		
Health Promotion Officer		
Student (PhD, Masters, Public Health Trainee)		
Other (please specify) ________________________________		

Q2.9 **How long had/have you been working in the primary position nominated above? (please circle one)**

Less than 6 months	6 months - 2 years
2 - 5 years	More than 5 years

Q2.10 **What is your professional background? (Select more than one if applicable)**

	**Approximate years of experience**
Medical Practitioner	
Aboriginal and/or Torres Strait Islander Health Practitioner/Worker	
Manager or Administrator	
Researcher	
Director (e.g. Board Director)	
Allied Health Professional	
Nurse	
Quality Improvement Facilitator	
Health Promotion Officer	
Policy or Planning Officer	
Other (please specify) ______________________________	

Q2.11 **Do you have expertise in a particular area relating to continuous quality improvement (CQI) in Indigenous primary health care?**

○ Yes (specify area of expertise)

____

○ No

Q2.12 **How long have/had you been involved with the CRE-IQI (established in December 2014)? (please circle one)**

Less than 6 months	6 months - 1 year	1 - 2 years
2 - 4 years	More than 4 years	

Q2.13 **Has your involvement in the CRE-IQI assisted you in your work, or in your health service? (please circle one)**

Yes	No

Q2.14 **Can you please state the most significant change that the CRE-IQI has made at each of these levels:**

For yourself:

____

____

____

____

____

For your team/work group**:**

____

____

____

____

____

For Indigenous primary health care services**:**

____

____

____

____

____

For the wider system level**:**

____

____

____

____

____

Q2.15 **Were you involved with the previous ABCD program of work (such as the ABCD National Research Partnership?) (please circle one)**

Yes	No	I don't know

Q2.16 **With which CRE-IQI Research or Work Programs have you been involved, or are you currently involved: (please circle all relevant responses)**

Aim 1: Refining and building new processes and tools	Aim 2: Improving data reporting systems	Aim 3: Improving use of QI data in clinical governance, management and practice
Aim 4: Building QI capacity in the Indigenous workforce	Aim 5: Monitoring and evaluating the impact of the CRE-IQI/Innovation Platform	Work Program 1: Promoting transfer of research outcomes into health policy/practice
Work Program 2: Developing the capacity of the health and medical research workforce	Work Program 3: Facilitating collaboration	None of these

Q2.17 **With which of these CRE-IQI Flagship projects have you been involved: (please circle all relevant responses)**

Engaging stakeholders in identifying priority evidence–practice gaps and strategies for improvement in primary health care (ESP project; Investigator: Prof. Ross Bailie)	Quality improvement in Aboriginal primary health care: Lessons from the best to better the rest (Investigator: Prof. Sarah Larkins)
Ongoing analysis and reporting of data from the ABCD National Research Partnership (Investigators: Prof. Ross Bailie and Dr Veronica Mathews)	Strategies for improving provision of maternal health care for Aboriginal and Torres Strait Islander women (Investigator: Dr. Melanie Gibson-Helm)
CQI approaches to sustainable implementation of social and emotional wellbeing programs and services (Investigator: Prof. Komla Tsey)	Monitoring and evaluation of the CRE-IQI as an innovation platform
None of these	

3. NETWORK

This section of the survey is for the social network analysis. For each CRE member in the list, please select the aspects that describe your interactions in the last 12 months using the categories below.

For each person, please nominate **in the last 12 months** whether you:

1) Knew this person before your initial participation in the CRE-IQI *(i.e. professionally or socially)*

2) Provided information to *(such as sharing of data, research articles, policy documents, or expert advice)*

3) Received information from *(such as sharing of data, research articles, policy documents, or expert advice)*

4) Have collaborated with on CRE-related research or project(s) *(you actively contributed to project tasks such as authored/co-authored publications or other project documents, procured funding or other resources, data collection or interviews, analytical work, etc)*

5) No involvement

6) This is me

Please select multiple categories per person as appropriate.

If you had no interaction or relationship with the person, you may mark “No involvement”. Please note the addition option to identify yourself where appropriate.

Q3.1 **For each person listed below, please select whether you knew, shared information and/or collaborated (see definitions above) with that person. Please select multiple categories per person as appropriate.**

	Please mark with ✓					
	**Knew prior to the CRE**	**Provided information to**	**Received information from**	**Collaborated with**	**No involvement**	**This is me**
e.g. John Smith, Employer Organisation (City)		✓		✓		
[CRE-IQI network member names and details have been deleted below for privacy requirements.]						

	Please mark with ✓					
	Knew prior to the CRE	Provided information to	Received information from	Collaborated with	No involvement	This is me

	Please mark with ✓					
	Knew prior to the CRE	Provided information to	Received information from	Collaborated with	No involvement	This is me

	Please mark with ✓					
	Knew prior to the CRE	Provided information to	Received information from	Collaborated with	No involvement	This is me

Q3.2 **Please nominate your relationships from the last 12 months with any additional participants of the CRE-IQI not listed in the previous section:**

We encourage you to freely nominate other CRE-IQI participants whom you have known, shared information with or collaborated with. These people may in-turn be invited to provide feedback on the CRE-IQI network if they haven't already done so. This process will provide a richer understanding of the network composition and dynamics, and of the reach and scale of the CRE-IQI network.

Nominate as many or as few people as you wish. If you wish to nominate more you may contact the survey team directly at boyd.potts@menzies.edu.au or append to this document.

All responses are in-confidence and will be de-identified for reporting. No identifiable information will be shared or reported.

	Please mark with ✓			
**Name of person, and workplace if known**	**Knew prior to the CRE**	**Provided information to**	**Received information from**	**Collaborated with**
e.g. John Smith, Employer Organisation		✓	✓	✓

Q3.3 **Please estimate the number of other people to whom you have disseminated research, findings or information from the CRE-IQI (e.g. through conferences, presentations, readership; as a guide, this number could be between 0 and 10,000)**

____

Q3.4 **How did you <u>primarily</u> come to be involved with the CRE-IQI?**

○ I was involved in a previous program of work with the research group (e.g. ABCD)

○ I was working for an organisation that was already involved (or later became involved)

○ I applied for an advertised position and followed a recruitment process

○ I was invited/introduced by an existing member (you may provide names if you wish) ____

○ Other (please specify) ____

Q3.5 **Briefly, what motivates you to participate in the CRE-IQI?**

____

____

____

____

____

Q3.6 **In your own experience, how effective were the following mechanisms in developing relationships with CRE-IQI participants?**

	**Please mark with ✓ (one response per row)**				
	**Very ineffective**	**Ineffective**	**Effective**	**Very effective**	**Don't know**
Attending face-to-face Biannual Meetings					
Attending CRE-IQI Masterclasses					
Direct introduction by another CRE-IQI participant in the usual course of my project work					
Actively seeking persons with specific skills or information directly related to my project					
Cold calling/emailing					
Direct introduction by another CRE-IQI participant outside the usual course of my project work					
Other (please specify) ______________________________ ______________					

4. YOUR EXPERIENCE OF THE CRE-IQI

This section of the survey provides feedback on the functioning and performance of the network with respect to the aims and principles of the CRE-IQI, how well it has achieved its goals, and its impacts in research and primary care.

We understand your ability to answer some items may depend on the level of your involvement. You may select "don't know" or pass items at any time, however all feedback is valued.

Q4.1 **How would you assess the CRE-IQI's level of achievement in meeting each of these goals:**

	**Please mark with ✓ (one response per row)**				
	**Very poor**	**Poor**	**Good**	**Very good**	**Don't know**
Refining and building new processes and tools					
Improving data reporting systems					
Improving use of QI data in clinical governance, management and practice					
Building QI capacity in the Indigenous workforce					
Monitoring and evaluating impact of the CRE-IQI					
Promoting transfer of research outcomes into health policy/practice					
Developing the capacity of the health and medical research workforce					
Facilitating collaboration					

Q4.2 **To what extent to you agree or disagree with each of these statements about *how the CRE-IQI works* most of the time:**

	**Please mark with ✓ (one response per row)**				
	**Strongly disagree**	**Disagree**	**Agree**	**Strongly agree**	**Don't know**
The CRE-IQI has a clear purpose and direction					
Participants understand the CRE-IQI goals					
Aboriginal and Torres Strait Islander people lead and direct the CRE-IQI research					
There is clear leadership of (or champions for) the CRE-IQI					
Only a few are involved in discussion, not everyone					
The CRE-IQI is hierarchically managed (top-down decision-making)					
My workplace is supportive of my involvement in the CRE-IQI					
CRE-IQI participants understand and are committed to CQI					
CRE-IQI meetings are well-organised and efficient					

Q4.3 **To what extent do you agree or disagree with each of these statements about *membership and involvement* in the CRE-IQI:**

	**Please mark with ✓ (one response per row)**				
	**Strongly disagree**	**Disagree**	**Agree**	**Strongly agree**	**Don't know**
People involved in the CRE-IQI trust each other					
The CRE-IQI is widely inclusive of people with different levels of professional seniority					
I have a lot of respect for the other people involved in the CRE-IQI					
The CRE-IQI actively supports Indigenous participation					
I have been able to trust the CRE-IQI participants from outside my own organisation to effectively contribute to project goals					
The CRE-IQI is widely inclusive in the range of professional backgrounds of people involved					

Q4.4 **To what extent do you agree or disagree with each of these following statements about *communication* in the CRE-IQI:**

	Please mark with ✓ (one response per row)				
	**Strongly disagree**	**Disagree**	**Agree**	**Strongly agree**	**Don't know**
The CRE-IQI has good communication and coordination with participants					
The CRE-IQI has facilitated inter-disciplinary collaboration amongst participants					
The CRE-IQI has not improved information sharing between participants					
The CRE-IQI disseminates its outputs widely in the area of CQI in Indigenous primary health care					

Q4.5 **To what extent do you agree or disagree with each of these statements in relation to CRE-IQI *effects or impacts*:**

	Please mark with ✓ (one response per row)				
	**Strongly disagree**	**Disagree**	**Agree**	**Strongly agree**	**Don't know**
I have acquired new knowledge and skills through the CRE-IQI					
I have built new formal relationships beneficial to my work					
I have built new informal relationships beneficial to my work					
The CRE-IQI has facilitated the effective use of data for quality improvement					
The CRE-IQI has facilitated acquisition of additional funding or other resources					
CRE-IQI collaboration has resulted in new research in CQI in Indigenous primary health care					
My time and effort spent with the CRE-IQI is worthwhile					

Q4.6 **In the *__last 12 months__*, to what extent has each of these been a barrier or a facilitator to the CRE-IQI's ability to improve CQI in Indigenous primary health care:**

	**Please mark with ✓ (one response per row)**				
	**Major barrier**	**Minor barrier**	**Minor facilitator**	**Major facilitator**	**Don't know**
Financial resources					
Human resources (e.g. staff, professional expertise)					
Physical resources (e.g. sites for meetings, education)					
Existing regulations and policies					
Infrastructure to collect and analyse data					
Attitudes and beliefs held by professionals and organisations					

Q4.7 **Have the following CRE-IQI materials and activities been useful to you in your work?**

	**Please mark with ✓ (one response per row)**			
	**Not at all useful**	**Moderately useful**	**Very useful**	**Extremely useful**
E-newsletters				
Website				
Reports				
Peer-reviewed publications				
Twitter				
CRE face-to-face Biannual Meetings				
Research Capacity Strengthening Teleconferences				
CRE Masterclasses				

Q4.8 **Please provide your suggestions (if any) for improving the effectiveness of the CRE-IQI as a collaborative network**
