# Supplementary material for: Network evaluation of an innovation platform in continuous quality improvement in Australian Indigenous primary healthcare
Source: Health Res Policy Syst. 2022 Oct 31;20:119. doi: 10.1186/s12961-022-00909-z (PMC9620635; doi:10.1186/s12961-022-00909-z)

Additional file 3.

Table S1 Definition of social network terms

Measure	Definition and measurement[a]
**Actors/Nodes**	Actors make up the 'social network'. They are represented by 'nodes' in network graphs, and can be individuals, groups, teams, communities, organisations, etc.
**Average clustering**	Clustering occurs when two actors have another mutual acquaintance, or several. Average clustering is measured by the average value of all individual clustering coefficients (excluding nodes with degree 1). Individual clustering coefficients measure the proportion of links between the neighbours of a given node, divided by the maximum number of links possible between them.
**Average degree**	Average degree is measured by the average number of connections (ties or edges) per node to other nodes in the network.
**Average path length**	A path is the number of edges between any node and any other node. The average path length represents the average path length between all pairs of nodes (popularised in the term "six degrees of separation", and known as the small-world phenomenon).
**Centralisation**	A measure of connectedness around the most central node (defined in this study by the highest node degree).
**Connected components**	Connected components refers to the number of subgroups in the network that are connected by at least one edge. Fewer connected components means more connectivity across the network. Isolated nodes (isolates) are counted as discrete components.
**Density**	Density reflects the extent to which network members are connected to each other (whether a network is dense or loose). Where ties are dense, information and influence can spread rapidly among all those who are in frequent contact. Density is defined as the number of edges present in the network, expressed as a proportion of the maximum possible number of edges.
**Directed network**	A directed network graph shows the direction of the connection between edges (ties), which are not necessarily mutual. Undirected network graphs, in contrast, contain relations that do not distinguish between senders and receivers.
**Edge, tie or link**	An edge, tie or link connects two nodes in a network.
**Network diameter**	The diameter of the network is the shortest distance between the two most distant nodes in the network. It is measured by the maximum number of edges needed to connect any two nodes in the network.
**Reciprocity (sharing)**	The proportion of two-way sharing (both provided and received information to/from the same person) out of all observed sharing in the network.
**Social network**	A social network consists of a set of nodes (actors) connected by some type of relations (also called edges, ties or links).
**Small-world network**	A network that exhibits a combination of short paths and social structure, the latter being defined in terms of network clustering.
**Trust**	Trust refers to the quality of the relationship among partners, that is, based solely on formal agreements, rules and procedures, or on trust and informal norms of reciprocity.
**Whole-network**	In whole-network studies, a researcher imposes a network boundary and focuses only on ties among actors in that population.

[a] Definition of terms is informed by Scott, J. Social network analysis: a handbook. Second Edition. London: Sage; 2000.

Table S2 Most significant change made by the CRE-IQI for respondents at four levels, 2017 and 2019

Most significant change reported by respondents	2017 Rank (n=48)	2019 Rank (n=39)
**1. Individual level**		
Increased networking / collaboration	1	1
Knowledge transfer and skills in CQI / capacity building	2	2
Assisting with progress in their careers / mentoring	3	3
Research capacity building	-	3
**2. Team or work group level**		
Increasing knowledge and skills in CQI / capacity building	1	2
Increased networking / collaboration	2	1
Grant support / development of new projects / health service support	-	3
**3. Primary health care service level**		
Increased knowledge and skills in CQI / capacity building	1	1
Research translation	2	-
Increased networking / collaboration	3	2
Grant support / development of new projects / health service support	-	1
**4. Wider system level**		
Research translation	1	-
Increased networking / collaboration	2	-
Access to information and data	3	-
Increased knowledge and skills in CQI / capacity building	4	1
Policy impact	-	2
Research evidence on CQI	-	3

Table S3 Members' overall perceptions of CRE-IQI, 2017 and 2019

Item	Most highly rated selections	2017 (n= 49) Mean of 4 point Likert scale	2019 (n=47) Mean of 4 point Likert scale
**Level of achievement in meeting CRE-IQI goals (2017: n=46, ~75.4% response: 2019: n=25-34, ~74.4% response)**	Facilitating collaboration	3.74	3.91
	Improving use of quality improvement data in clinical governance, management and practice	3.20	3.41
	Monitoring and evaluating the impact of the CRE-IQI	3.17	3.75
	Developing the capacity of the health and medical research workforce	3.17	3.33
	Promoting transfer of research outcomes into health policy/practice	3.11	3.32
	Building quality improvement capacity in indigenous workforce	3.09	3.45
	Refining and building new processes and tools	3.02	3.21
	Improving data reporting systems	2.98	3.04
**How the CRE-IQI functions (2017: n=46, 75.4% response; 2019: n=35-38, ~90.1 response)**	Clear leadership of (or champions for) the CRE-IQI	3.59	3.66
	CRE-IQI participants understand and are committed to CQI	3.54	3.57
	CRE-IQI meetings are well-organised and efficient	3.46	3.89
	My workplace is supportive of my involvement in the CRE-IQI	3.33	3.61
	The CRE-IQI has a clear purpose and direction	3.17	3.47
	CRE-IQI participants understand the CRE goals	3.13	3.25
	Indigenous people lead and direct the CRE-IQI research[a]	n.a.	3.00
**Membership and involvement in the CRE-IQI (2017: n=45, 73.8% response; 2019: n=33, ~92.1% response)**	The CRE-IQI actively supports Indigenous participation	3.38	3.63
	People involved in the CRE-IQI trust each other	3.24	3.43
	The CRE is widely inclusive in the range of professional backgrounds of people involved	3.18	3.59
	I have a lot of respect for the other people involved in the CRE-IQI[a]	n.a.	3.84
	I have been able to trust the CRE-IQI participants from outside my own organisation to effectively contribute[a]	n.a.	3.73
**Communication in the CRE-IQI (2017: n=45, 73.8% response;**	The CRE-IQI has good communication with participants	3.40	3.62
	The CRE-IQI has facilitated inter-disciplinary collaboration amongst participants	3.31	3.51

**2019: n=31-37, ~88.1% response)**	The CRE-IQI distributes its outputs widely in the area of CQI in Indigenous PHC	3.07	3.42
**Perceptions of CRE-IQI outcomes and impacts (2017: n=46, 75.4% response; 2019: n=29-38, ~88.2% response)**	My time and effort spent with the CRE-IQI is worthwhile	3.52	3.76
	I have built new informal relationships beneficial to my work	3.48	3.74
	I have acquired new knowledge and skills through the CRE-IQI	3.41	3.58
	I have built new formal relationships beneficial to my work	3.39	3.61
**Perceptions of facilitators for the CRE-IQI (2017: n=42, 68.9% response; 2019: n=19-28, ~55.8% response)**	Human resources (e.g. staff, professional expertise)	2.95	3.27
	Physical resources (e.g. sites for meetings, education)	2.79	3.39
	Attitudes and beliefs held by professionals and organisations	2.62	3.18
**Effectiveness of mechanisms in developing relationships with CRE-IQI participants (2019: n=34-38, ~66.0% response)**	Attending face-to-face Biannual Meetings[a]	n.a.	3.71
	Direct introduction by another CRE-IQI participant in the usual course of my project work[a]	n.a.	3.32
	Attending CRE-IQI Masterclasses[a]	n.a	3.32
**Usefulness of CRE-IQI materials (2017: n=49, 80.3% response; 2019: n=34-38, ~89.7% response)**	Publications	2.82	2.95
	Reports	2.63	2.86

[a] Six additional items were included in the 2019 survey.

Table S4 Degree centralisation of networks, 2017 and 2019

	Degree centralisation	
**Relationship**	**2017**	**2019**
Knew Previously	0.54	0.41
Collaborated	0.77	0.61
Shared Information	0.63	0.62

Table S5 Community detection in networks, 2017 and 2019

Timepoint	Relationship	Communities detected	Attribute	X2	df	p	Cramer's V	Effect size
2017	Knew Previously	5	Organisation type	22.15	20	0.33	0.26	Moderate
2017	Knew Previously	5	Primary work role	24.29	20	0.23	0.27	Moderate
2017	Collaborated	3	Organisation type	30.98	10	<.001	0.44	Moderate
2017	Collaborated	3	Primary work role	25.04	10	<.01	0.39	Moderate
2017	Shared Information	4	Organisation type	18.61	15	0.23	0.28	Moderate
2017	Shared Information	4	Primary work role	13.92	15	0.53	0.24	Moderate
2019	Knew Previously	17	Organisation type	144.78	80	<.001	0.60	Moderate
2019	Knew Previously	17	Primary work role	126.21	80	<.001	0.56	Moderate
2019	Collaborated	6	Organisation type	23.76	25	0.53	0.24	Moderate
2019	Collaborated	6	Primary work role	36.77	25	0.06	0.30	Moderate
2019	Shared Information	5	Organisation type	32.84	20	0.04	0.32	Moderate
2019	Shared Information	5	Primary work role	33.47	20	0.03	0.32	Moderate

Figure S1 Degree distribution for network of prior knowledge, 2017 and 2019

2017

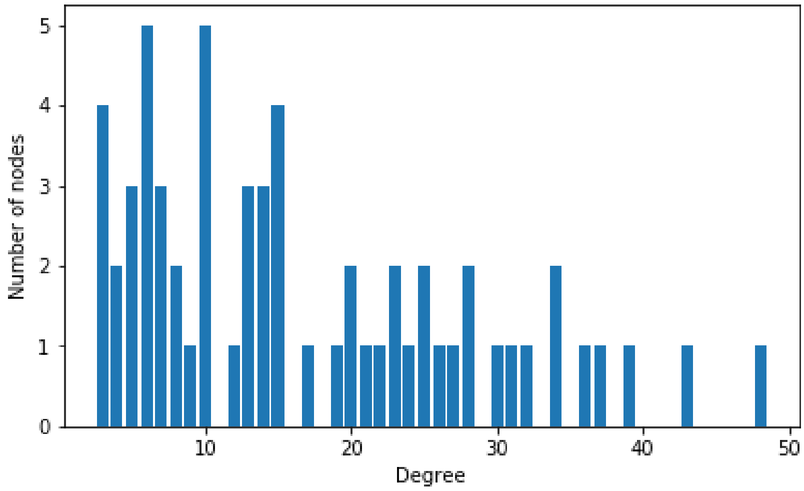

2019

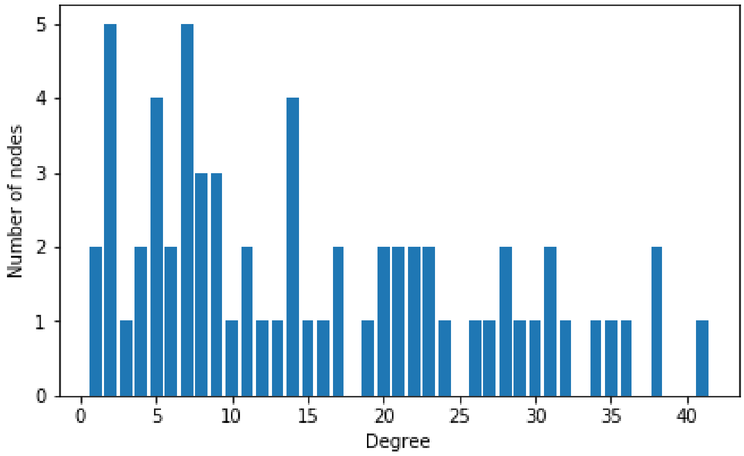

Figure S2 Degree distribution for network of information sharing, 2017 and 2019

2017

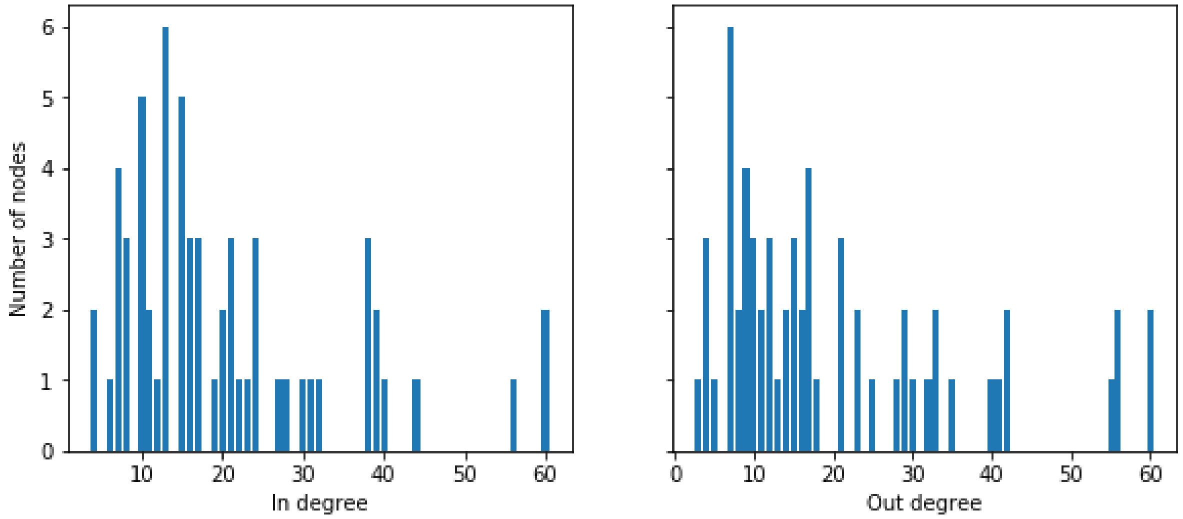

2019

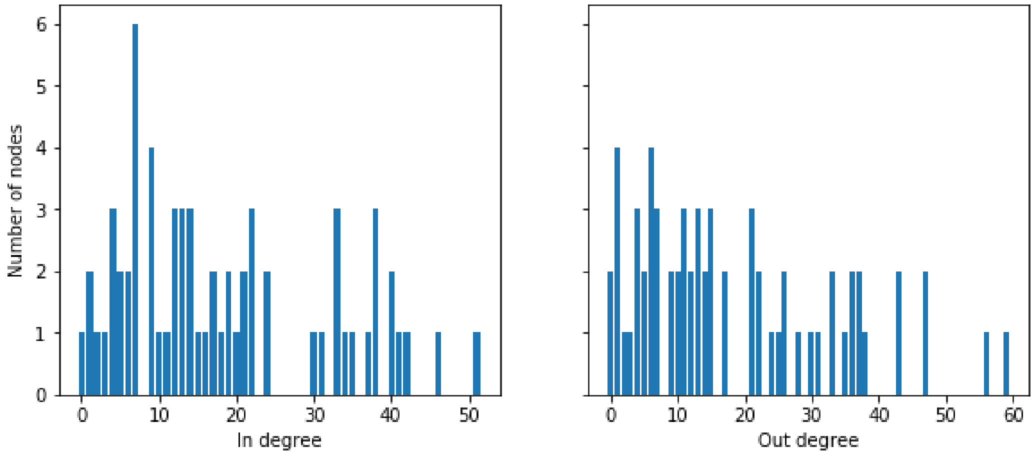

Figure S3 Degree distribution for network of collaborations, 2017 and 2019

2017

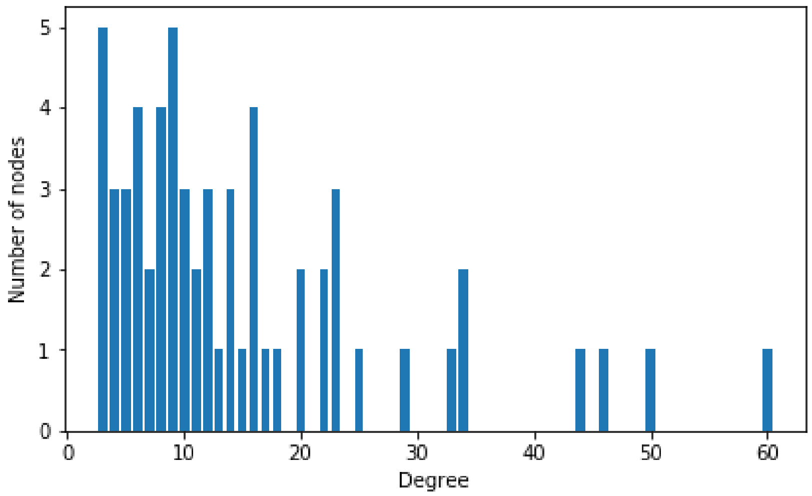

2019

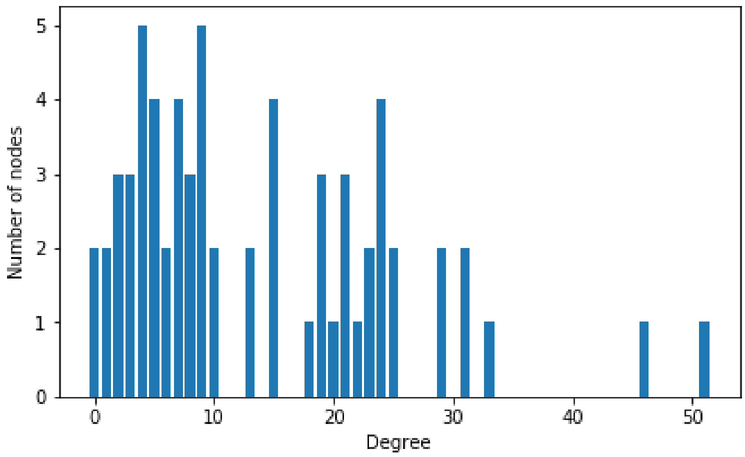

Supplement: Supplementary file 3 — Additional file 3: Table S1. Definition of social network terms; Table S2. Most significant change made by the CRE-IQI for respondents at four levels, 2017 and 2019; Table S3. Members’ overall perceptions of CRE-IQI, 2017 and 2019; Table S4. Degree centralization of networks, 2017 and 2019; Table S5. Community detection in networks, 2017 and 2019; Figure S1. Degree distribution for network of prior knowledge, 2017 and 2019; Figure S2. Degree distribution for network of information-sharing, 2017 and 2019; Figure S3. Degree distribution for network of collaborations, 2017 and 2019. [file 12961_2022_909_MOESM3_ESM.pdf]
